# P200 family protein IFI204 negatively regulates type I interferon responses by targeting IRF7 in nucleus

**DOI:** 10.1371/journal.ppat.1008079

**Published:** 2019-10-11

**Authors:** Liu Cao, Yanxi Ji, Lanyi Zeng, Qianyun Liu, Zhen Zhang, Shuting Guo, Xiaolong Guo, Yongjia Tong, Xiaolu Zhao, Chun-Mei Li, Yu Chen, Deyin Guo

**Affiliations:** 1 State Key Laboratory of Virology, Modern Virology Research Center, College of Life Sciences, Wuhan University, Wuhan, China; 2 School of Medicine, Sun Yat-sen University, Guangzhou, China; 3 School of Basic Medical Sciences, Wuhan University, Wuhan, China; 4 College of Life Sciences, Wuhan University, Wuhan, China; University of Southern California, UNITED STATES

## Abstract

Interferon-inducible p200 family protein IFI204 was reported to be involved in DNA sensing, and subsequently induces the production of type I interferons and proinflammatory mediators. However, its function in the regulation of antiviral innate immune signaling pathway remains unclear. Here we reported a novel role of IFI204 that specifically inhibits the IRF7-mediated type I interferons response during viral infection. IFI204 and other p200 family proteins are highly expressed in mouse hepatitis coronavirus-infected bone marrow-derived dendritic cells. The abundant IFI204 could significantly interact with IRF7 in nucleus by its HIN domain and prevent the binding of IRF7 with its corresponding promoter. Moreover, other p200 family proteins that possess HIN domain could also inhibit the IRF7-mediated type I interferons. These results reveal that, besides the positive regulation function in type I interferon response at the early stage of DNA virus infection, the interferon-inducible p200 family proteins such as IFI204 could also negatively regulate the IRF7-mediated type I interferon response after RNA virus infection to avoid unnecessary host damage from hyper-inflammatory responses.

## Author summary

The regulation of type I interferon signaling pathway is dynamic sequential processes and must be tightly regulated to keep balance between antiviral immune and hyper-inflammatory responses. The precise regulation mechanisms of the innate immune signaling pathway are still worth studying. Here, we found a novel role of the interferon-inducible p200 family protein IFI204 that specifically inhibits the IRF7-mediated type I interferon production by negative control of the transcriptional activity of IRF7 in the nucleus at the late stage of RNA virus infection. Previous studies showed that IFI204 is involved in DNA sensing during DNA virus infection to initiate antiviral immune responses. We demonstrate that IFI204 can inhibit IRF7-mediated activation of type I IFN responses induced by RNA virus infection, which is in contrast with its role in IRF3 activation in cGAS-STING DNA sensing pathway during DNA virus infection. Such negative regulation may help to avoid hyper-inflammatory responses induced by the over-activated IRF7-mediated type I interferons at late stage of the viral infection. Thus, the current study sheds light on the regulation roles of p200 family proteins and the accurate regulation system of type I interferons signaling pathway.

## Introduction

The interferon (IFN)-mediated innate immune response is important for the control of viral infection by the effect of downstream IFN-stimulated genes (ISGs) [1]. It is initiated from the recognition of pathogen-associated molecular patterns (PAMPs) by host pattern-recognition receptors (PRRs). For examples, the viral RNA is recognized by retinoic acid-inducible gene I (RIG-I)-like receptors (RLRs); the viral DNA is detected by cytosolic DNA sensors including cyclic GMP-AMP synthase (cGAS) and AIM2-like receptors (ALRs) [1–6].

Recognition by different PRRs activates the expression of type I IFNs (IFNα and IFNβ) via different signaling pathways, triggering IFN regulatory factors (IRFs)-mediated transcriptions. RLRs, including RIG-I, MDA5 and LGP2, mainly recognize viral dsRNA or ssRNA, and ultimately lead to MAVS-IRF3-mediated transcription of target genes and the production of type I IFNs [7–9]. There are more than ten TLRs, three of which can recognize viral RNAs in endosome [10–13]. TLR3 recognizes dsRNA and recruits the adaptor molecule TRIF to promote IRF3/7 import into the nucleus to activate the expression of type I IFNs and ISGs [14, 15]; TLR7/8 detect ssRNA and recruit the adaptor molecule myeloid differentiation primary-response protein 88 (MyD88) to activate IRF7 to trigger the expression of type I IFNs [16, 17]. Both of the cytoplasmic DNA sensor cGAS and ALRs, including AIM2, IFI16 and IFI204, can activate the STING-IRF3 signaling pathway to induce the type I IFNs [4, 18–21].

The ALRs belong to the interferon-inducible p200 family proteins (also known as PYHIN or HIN-200 proteins), which contains a pyrin (PYD) domain at the amino-terminus and at least one DNA binding HIN domain at carboxy-terminus [22, 23]. Among the p200 family proteins, IFI16, AIM2 and MNDA are from humans, and IFI204, IFI205, IFI209, AIM2, MNDA and MNDAL are from mice. In addition to their functions in DNA sensing pathways [21, 24–30], the p200 family proteins can also act as modulators of many cellular functions, including cell proliferation, differentiation, apoptosis, senescence and DNA repair [23, 31]. In addition, IFI16 has been identified as an ISG that triggers proinflammatory mediators and therefore plays an important role in autoimmune diseases [26, 32]. However, Gray and colleagues have reported that ALRs including 13 PYHIN proteins are dispensable for interferon response to viral DNA in mice [33]. Therefore, the functions of p200 family proteins in antiviral responses need to be further clarified.

In this study, we demonstrated that the p200 family proteins, represented by IFI204, which is well known as an ALR and murine ortholog of IFI16 [28, 34], were markedly induced in bone marrow-derived dendritic cells (BMDCs) after infection by mouse hepatitis coronavirus (MHV). Interestingly, we found that the IFI204 can specifically interact with IRF7 with its HIN domain and therefore hijack IRF7 in nucleus, consequently blocking the binding of IRF7 with its cognate transcriptional promoters. Moreover, other p200 family proteins that harbor a HIN domain could also bind with IRF7 and specifically down regulate the downstream transcription. Our results reveal a novel mechanism of negative regulation of type I IFNs response by p200 family proteins during RNA virus infection, which may prevent the host innate immune system from over-activation. This indicates that ALRs play two opposite roles during the infection of DNA and RNA viruses.

## Results

### Higher expression of P200 family protein IFI204 is induced in BMDCs by MHV infection

**Fig 1:**
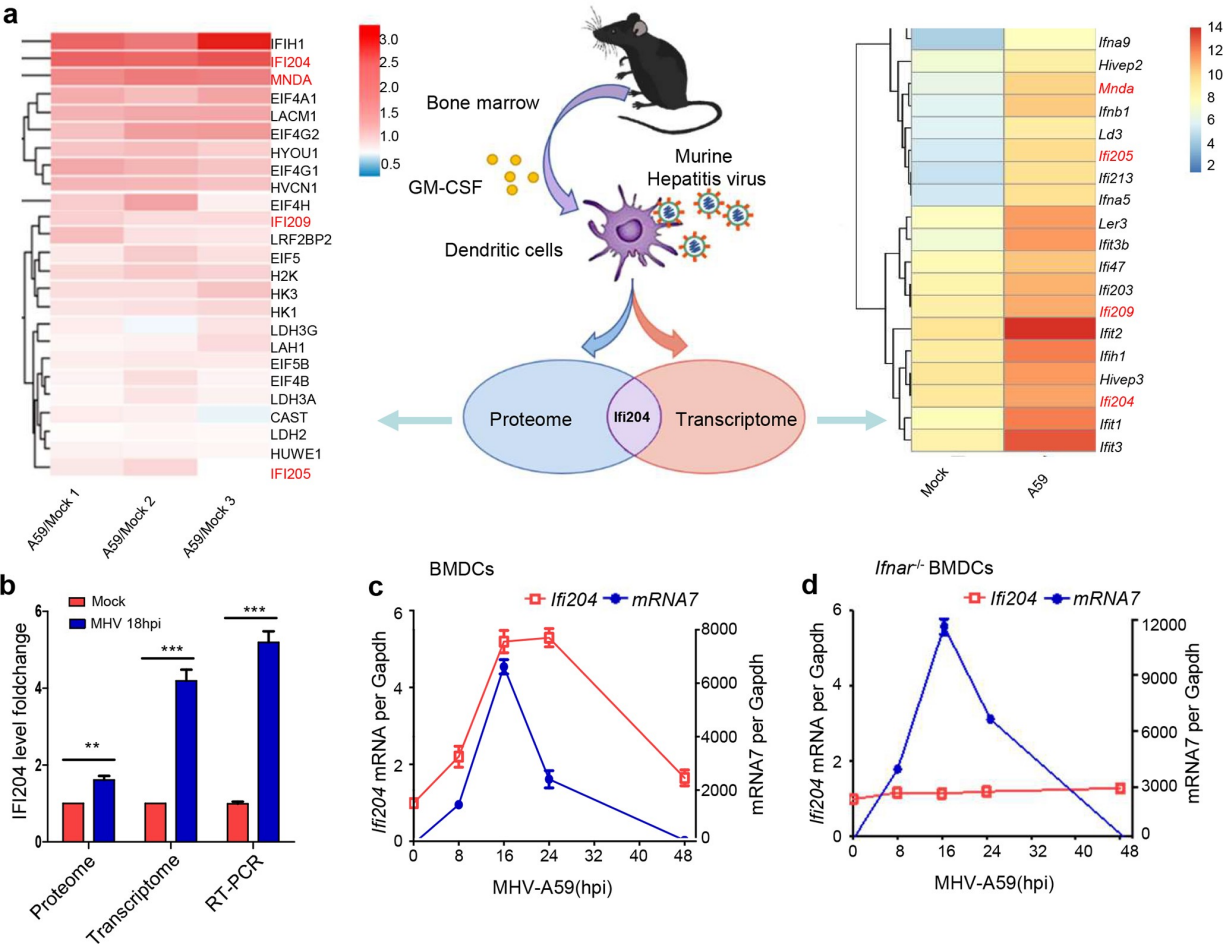
P200 family protein IFI204 is highly expressed in MHV-infected BMDCs. (A) Schematic of the experiment. Bone marrow cells were isolated from mouse tibia and femur and cultured for 7 to 9 days in medium containing mouse GM-CSF. BMDCs were infected by MHV (MOI = 1) and collected at 18 hpi. The cells were collected and examined for transcriptome and proteome. The graph (right panel) shows the average value of three independent experiments of transcriptome. (B) Proteome, transcriptome and qRT-PCR analyses identified higher expression of *Ifi204* in MHV-infected BMDCs at 18 hpi. (C and D) qRT-PCR analysis of *Ifi204* and MHV *mRNA7* in MHV-infected BMDCs (C) and *Ifnar*^-/-^ BMDCs (D) at different time points as indicated. **P < 0.01 and ***P < 0.001. Data are representative of three independent experiments (mean ± SD in B).

To identify the function of various cell factors induced by RNA virus infection, we adopted a BMDC infection model by murine hepatitis virus (MHV), which is typical RNA virus with positive single stranded RNA. As shown in Fig 1A, bone marrow cells were harvested from 8-week-old mice (C57BL/6) and induced with GM-CSF for 7–8 days. BMDCs were infected by MHV and collected at 18 hours (hrs) post infection (hpi). The differential expression level of host factors was measured by analyzing the transcriptome and proteome before and after MHV infection. We found that over 1000 genes at mRNA level and 200 at protein level were changed, among which 100 cellular factors were up-regulated or down-regulated both at mRNA and protein levels (Fig 1A). In this model, the transcription of *Ifnb*, *Ifna1*, *Ifna4*, *Ifna6*, *Tnf* and *Il6* were significantly activated (S1 Fig), indicating that the IFN response and proinflammatory cytokines were apparently induced. Notably, the p200 family proteins, including IFI204, IFI205, IFI209 and MNDA were expressed at higher level after MHV infection (Fig 1A). Interestingly, IFI204, which is previously reported to initiate type I IFNs in DNA virus-infected cells, obtained high scores as up-regulated factor both at mRNA and protein levels (Fig 1B). Quantitative RT-PCR (qRT-PCR) analysis confirmed that the MHV infection induced a 5-fold upregulation of the mRNA of *Ifi204* at 18 hpi (Fig 1B). Moreover, the transcriptional regulation of Ifi204 is consistent with viral growth curve in MHV-infected BMDCs (Fig 1C). However, Ifi204 was not stimulated in IFN-associated receptor-deficient (*Ifnar*^-/-^) BMDCs (Fig 1D), indicating that the upregulation is interferon-dependent. Furthermore, the similar phenomena were observed in wt BMDCs and *Ifnar*^-/-^ BMDCs infected by a DNA virus (HSV-1) (S2 Fig). These data indicate that the expression of IFI204 is induced by type I IFNs during the infections of DNA or RNA viruses.

### IFI204 inhibits RNA virus-triggered type I IFNs responses

**Fig 2:**
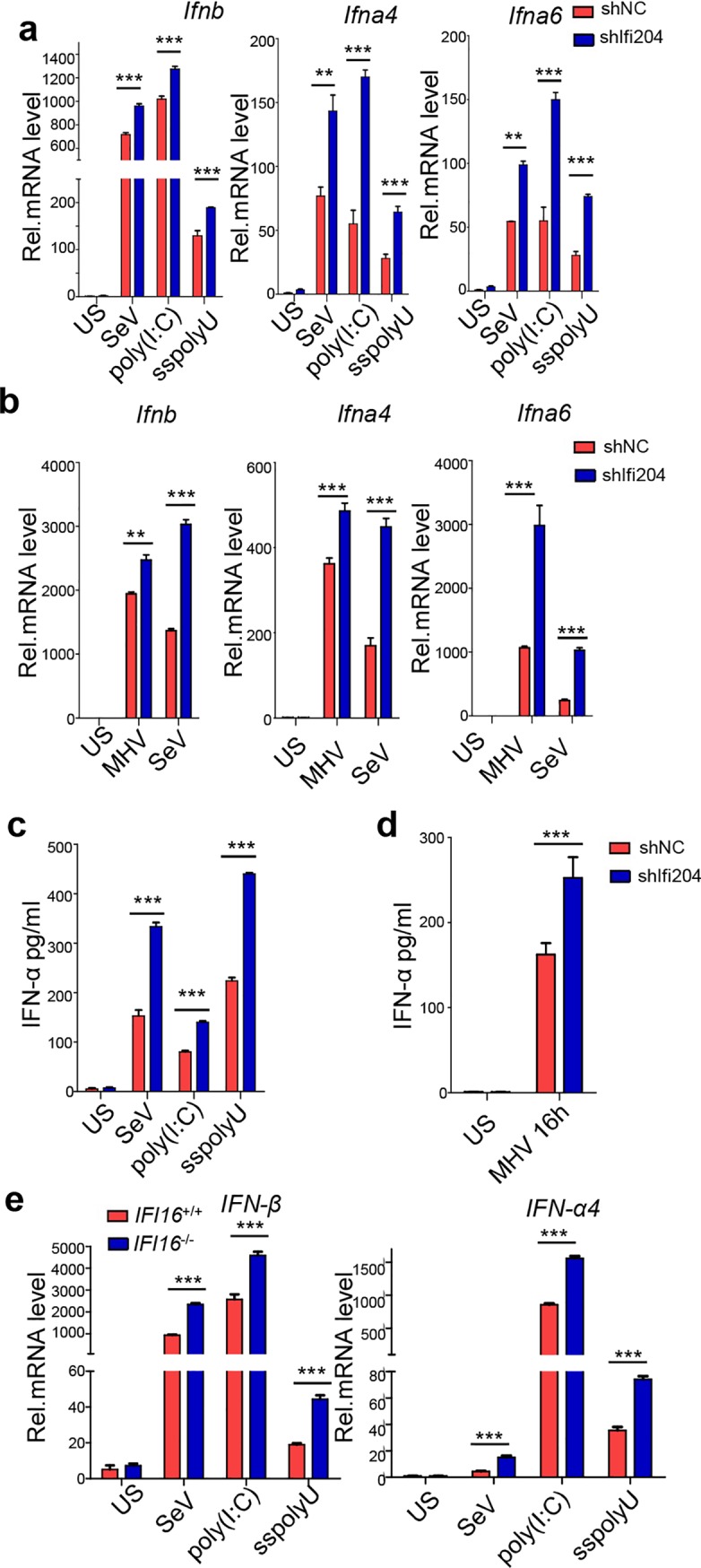
IFI204 inhibits the production of type I IFNs in RNA virus-infected cells. (A) qRT-PCR analysis of *Ifnb*, *Ifna4* and *Ifna6* in NIH3T3 cells with stable knockdown of IFI204 by lentivirus-mediated shIFI204. The cells were unstimulated (US) or stimulated with SeV, poly(I:C) (10 μg/ml) or ssPolyU (10 μg/ml) as indicated for 8 hrs. (B) qRT-PCR analysis of *Ifnb*, *Ifna4* and *Ifna6* in BMDCs with knockdown of IFI204 by lentivirus-mediated shIFI204. The cells were unstimulated (US) or stimulated with MHV (MOI = 1) or SeV as indicated for 8 hrs. (C and D) ELISA analysis of the production of IFNα in IFI204-knockdown NIH3T3 cells (C) and BMDCs (D). (E) qRT-PCR analysis of *Ifnb* and *Ifna4* in *IFI16*^-/-^ and *IFI16*^+/+^ A549 cells after stimulation with SeV, poly(I:C) (10 μg/ml) or ssPolyU (10 μg/ml) for 8 hrs. US, unstimulated control. **P < 0.01 and ***P < 0.001. Data are representative of three independent experiments (mean ± SD in A-E).

To investigate the role of IFI204 in RNA virus-triggered signaling, we designed three siRNAs targeting *Ifi204* (S3A and S3B Fig), the one of which being further packaged into lentivirus-mediated small hairpin RNA (shRNA) vector to generate an NIH3T3 cell line and BMDCs with stable IFI204 knockdown (S3C and S3D Fig). The qRT-PCR analysis suggested that knockdown of IFI204 stimulated Sendai virus (SeV)-, poly(I:C)- or ssPolyU-induced transcription of *Ifnb*, *Ifna4* and *Ifna6* in NIH3T3 cells (Fig 2A), MHV- or SeV-induced transcription of *Ifnb*, *Ifna4* and *Ifna6* in BMDCs (Fig 2B), and vesicular stomatitis virus (VSV)-induced transcription of *Ifnb*, *Ifna1* and *Ifna4* in RAW264.7 cells (S4 Fig). In addition, the production of IFNα was also significantly enhanced by knockdown of IFI204 in NIH3T3 cells (Fig 2C) and BMDCs (Fig 2D).

To confirm the similar function of IFI16, which is the human ortholog of IFI204, we generated *IFI16*^-/-^ A549 cell line by CRISPR/Cas9-mediated genome editing (S3E and S3F Fig). Consistent with the analysis in murine cells, the knockout of *IFI16* stimulated SeV-, poly(I:C)- or ssPolyU-induced transcription of *IFNβ* and *IFNα4* in A549 cells (Fig 2E). These data indicate that negative regulation of RNA virus-triggered type I IFNs by ALRS is conserved in both mouse and human cells.

Furthermore, previous studies reported that IFI16 acts on STING and cooperates with cGAS-STING pathway to promote the type I IFNs during the DNA virus infection [28, 29, 35]. To further clarify the function of IFI204 during the infection of DNA virus, we infected NIH3T3 cells with HSV-1. The qRT-PCR analysis revealed that knockdown of IFI204 and double knockdown of IFI204-STING or IFI204-cGAS significantly inhibited HSV-1-induced transcription of *Ifnb* to different degrees in NIH3T3 cells (S5A Fig). Moreover, we also observed consistent phenomena in HSV-1-infected A549 cells with *IFI16*^-/-^, *IFI16*^-/—^STING knockdown or *IFI16*^-/—^cGAS knockdown (S5B Fig), indicating that IFI204 might facilitate cGAS-STING DNA sensing pathway that leads to IRF3 activation during the infection of HSV-1. These data collectively suggest that IFI204 inhibits RNA virus-triggered type I IFNs, while promotes DNA virus-triggered type I IFNs.

### IFI204 inhibits IRF7-mediated activation of type I IFNs responses

**Fig 3:**
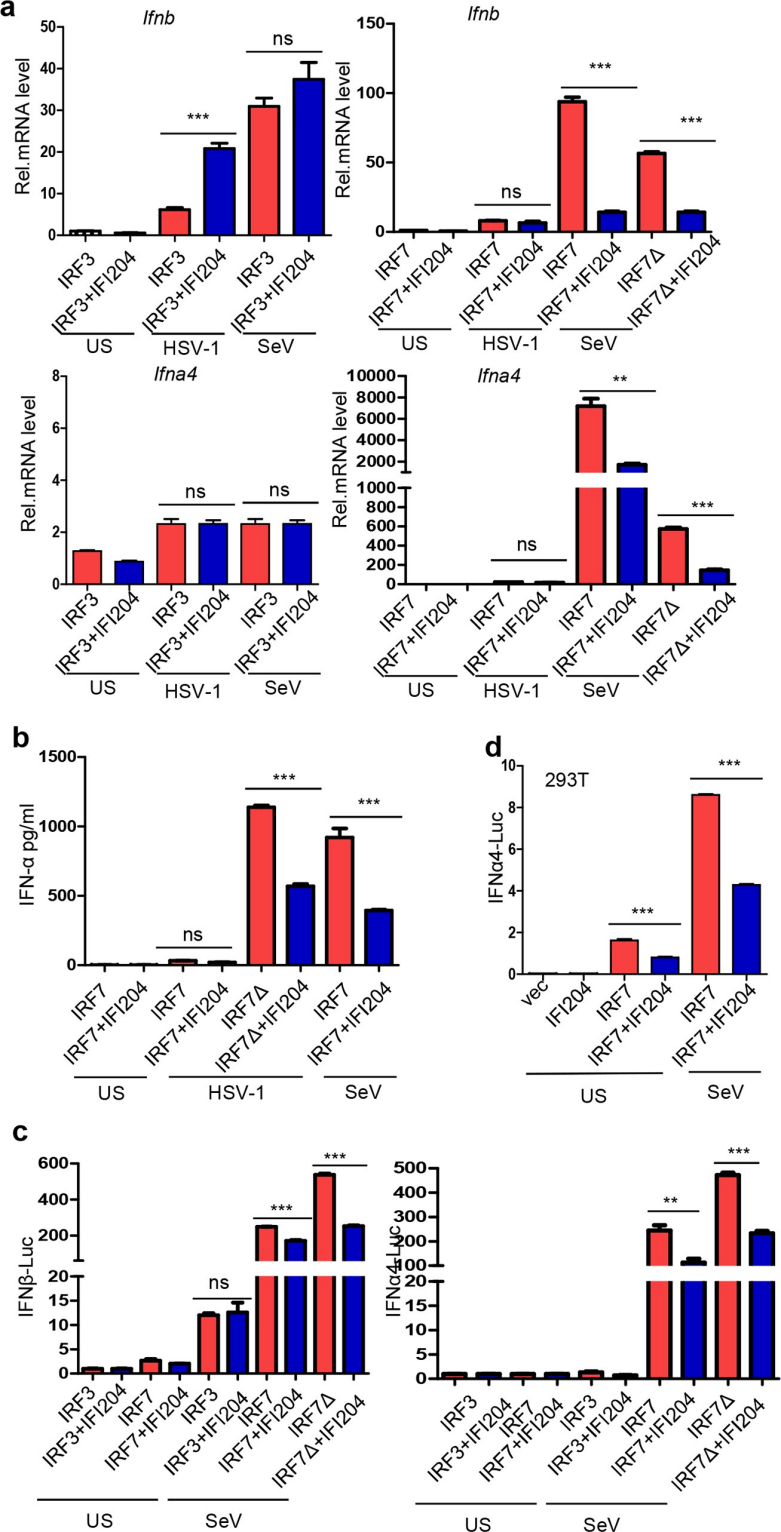
IFI204 inhibits IRF7-mediated activation of type I IFNs responses. (A) qRT-PCR analysis of *Ifnb* and *Ifna4* in *Irf3*^*−/−*^*Irf7*^*−/−*^MEFs transfected with IFI204, IRF3, IRF7 or IRF7-Δ247–467 mutant (IRF7Δ) as indicated. 24 hrs post-transfection, cells stimulated with HSV-1 (MOI = 1) or SeV for 12 hrs as indicated. The unstimulated (US) treatment was used as a control. (B) ELISA analysis of the expression of IFNα in *Irf3*^*−/−*^*Irf7*^*−/−*^MEFs transfected with IFI204, IRF7 or IRF7-Δ247–467 mutant (IRF7Δ) as indicated. 24 hrs post-transfection, cells were stimulated with HSV-1 (MOI = 1) or SeV for 12 hrs as indicated. The unstimulated (US) treatment was used as a control. (C) Dual luciferase assays for analyzing the promoter activity of IFNβ (left panel) and IFNα4 (right panel) in *Irf3*^*−/−*^*Irf7*^*−/−*^MEFs co-transfected with 200 ng IFNβ or IFNα4 promoter luciferase reporter plasmids and 250 ng IFI204, IRF3, IRF7 or IRF7-Δ247–467 mutant (IRF7Δ) as indicated. The Renilla expression plasmid (pRL-TK, 10 ng) was co-transfected as an internal control. 24 hrs post-transfection, cells were stimulated with SeV for 12 hrs as indicated. The unstimulated (US) treatment was used as a control. (D) Dual luciferase assays for analyzing the promoter activity of IFNα4 in HEK293T cells co-transfected with 200 ng IFNα4 promoter luciferase reporter plasmid, 250 ng IFI204 and IRF7 as indicated. The Renilla expression plasmid (pRL-TK, 10 ng) was co-transfected as an internal control. 24 hrs post-transfection, cells were stimulated with SeV for 12 hrs as indicated. The unstimulated (US) treatment was used as a control. Not significant (ns), **P < 0.01 and ***P < 0.001. Data are representative of three independent experiments (mean ± SD in A-D).

As is known, RNA viruses trigger IRF3- and IRF7-mediated activation of type I IFNs, while DNA viruses activate STING-IRF3 signaling pathway [24]. In our MHV-induced BMDCs model, a set of host factors in both IRF3 and IRF7 signaling pathway were stimulated (S6 Fig). Therefore, to find out which signaling pathway is inhibited by IFI204, IRF3 or IRF7 were co-transfected with IFI204 into *Irf3*^*−/−*^*Irf7*^*−/−*^ mouse embryonic fibroblasts (MEFs), which were infected by HSV-1 or SeV as the model of DNA or RNA virus, respectively. As shown in Fig 3A, in IRF3-transfected *Irf3*^*−/−*^*Irf7*^*−/−*^ MEFs, the transcription of both *Ifnb* and *Ifna4* could be activated by either HSV-1 or SeV infection. However, IFI204 can only enhance the transcription of *Ifnb* induced by the infection of HSV-1 in the presence of IRF3 as previously reported. In contrast, in IRF7-transfected *Irf3*^*−/−*^*Irf7*^*−/−*^ MEFs, the transcription of both *Ifnb* and *Ifna4* could only be activated by SeV infection or by the self-activating IRF7 mutant IRF7-Δ247–467 [36] (Fig 3A). Intriguingly, IFI204 significantly inhibited the transcription of both *Ifnb* and *Ifna4* in SeV-infected IRF7-rescuing MEFs or IRF7-Δ247-467-rescuing MEFs (Fig 3A). In addition, the ELISA analysis showed that the production of IFNα was also remarkably inhibited by IFI204 in SeV-infected IRF7-transfected *Irf3*^*−/−*^*Irf7*^*−/−*^ MEFs (Fig 3B). Consistent with Fig 3A, the infection of HSV-1 could not effectively induce the expression of IFNα in IRF7-rescuing MEFs. However, IFI204 could still inhibit the expression of IFNα induced by the self-activating IRF7-Δ247–467 mutant in the context of HSV-1 infection (Fig 3B). To confirm the above observed results, we further evaluated the transcriptional activation of *Ifnb* and *Ifna4* promoters through more sensitive luciferase assay in *Irf3*^*−/−*^*Irf7*^*−/−*^ MEFs. As shown in Fig 3C, IFI204 inhibited the transcriptional activation of *Ifnb* and *Ifna4* promoters in SeV-infected IRF7-rescuing and IRF7-Δ247-467-rescuing MEFs but not IRF3-rescuing MEFs. Furthermore, IFI204 could repress the transcriptional activation of *Ifna4* promoters induced by overexpression of IRF7 with or without SeV-infection in HEK293T cells (Fig 3D), indicating that the inhibition takes place at the downstream of IRF7 activation. Together, these results indicate that IFI204 can significantly inhibit IRF7-mediated type I IFNs responses, and this inhibition event only occurs in RNA virus-infected cells, which could effectively induce the IRF7-mediated signaling pathway.

### HIN domain of IFI204 interacts directly with IRF7

**Fig 4:**
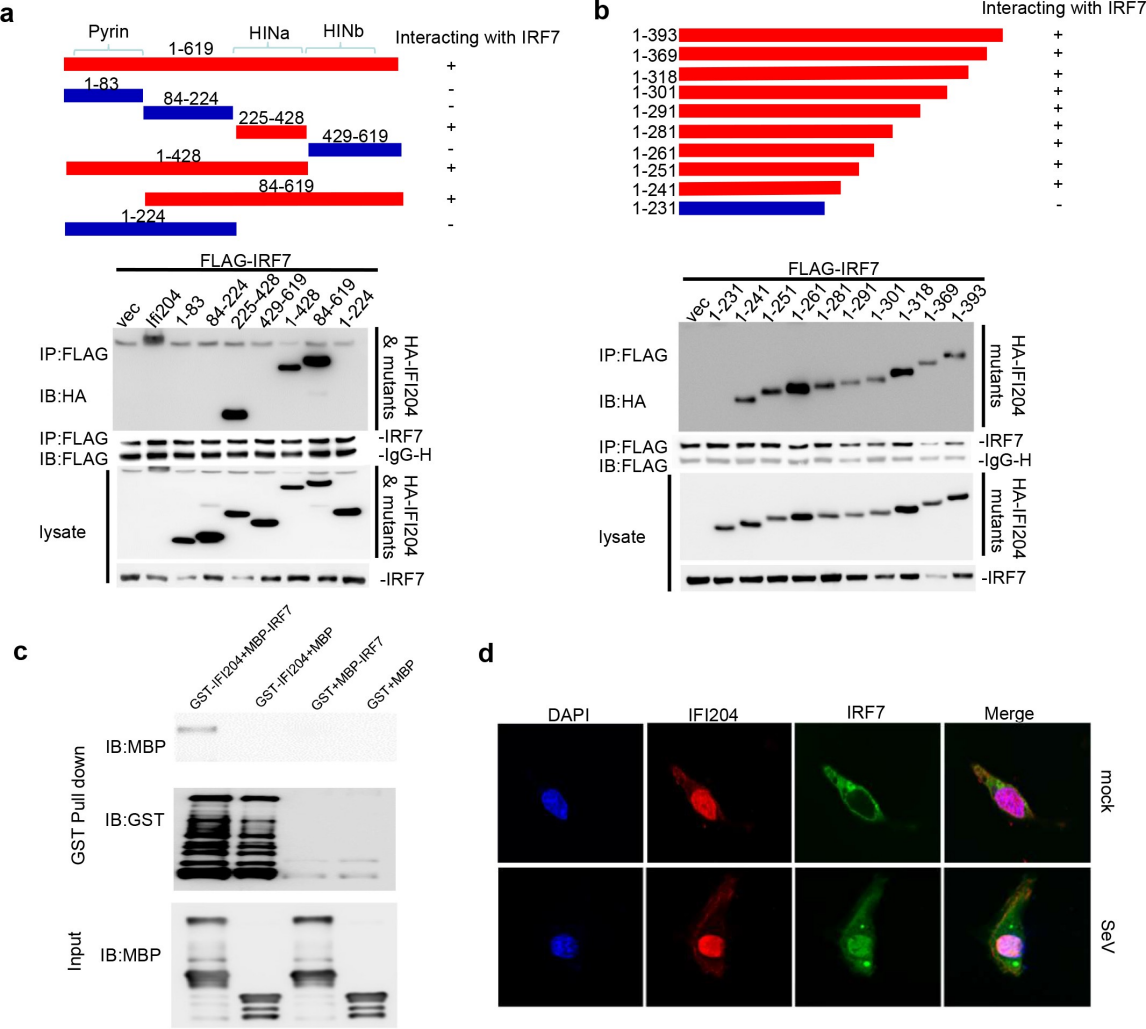
IFI204 interacts with IRF7. (A and B) Schematic diagram to show the regions of IFI204 interacting with IRF7 (upper panels in A and B). Co-immunoprecipitation (IP, with anti-Flag) and immunoblot (IB, with anti-HA) analysis of HEK293T cells that were transfected with plasmids encoding Flag-IRF7 and HA-IFI204 or its mutants. Cell lysate was analyzed by immunoblot with anti-Flag and anti-HA (lower panels in A and B). (C) GST pull-down assay. Purified GST-IFI204 or GST protein was incubated with MBP-IRF7 or MBP as indicated and pulled down with glutathione resin beads. Each sample was detected by Western blotting with the indicated antibodies. GST and MBP proteins were used as a negative control, respectively. (D) Intracellular localization of IFI204 and IRF7 assessed by immunostaining followed by laser scanning confocal microscopy. *Irf3*^*−/−*^*Irf7*^*−/−*^MEFs cells were co-transfected with pFlag-IRF7 and pHA-IFI204 stimulated with SeV for 0 h (mock) and 6 hrs (SeV) at 24 hrs post-transfection. Then cells were washed with PBS and harvested for immunofluorescence analysis. Mouse anti-HA antibody was used as primary antibody and CY3-conjugated goat anti-mouse IgG was used to detect IFI204 as shown in red fluorescence. Rabbit anti-flag antibody was used as primary antibody and FITC-conjugated goat anti-rabbit IgG was used to detect IRF7 as shown in green fluorescence. DAPI (4, 6-diamidino-2-phenylindole) is used for nuclei staining as shown in blue fluorescence. Data are representative of three independent experiments.

To explore the mechanism, we demonstrated that IFI204 could interact with IRF7 by exogenous and endogenous co-immunoprecipitation and immunoblot analysis (S7 Fig). Domain mapping analysis suggested that the first HIN domain (HINa) of IFI204 (aa 225–428) was responsible for the association (Fig 4A). Truncation mapping analysis showed that the N-terminus of HINa domain (aa 225–241) of IFI204 and the DBD domain (1-134aa) of IRF7 were the key interaction motifs (Fig 4B and S8 Fig). To test whether it is direct association between IFI204 and IRF7, GST-fused IFI204 and MBP-fused IRF7 were expressed and purified in vitro, and the GST pull-down analysis suggested that the association between IFI204 and IRF7 is direct interaction (Fig 4C). To confirm the interaction in vivo, the IFI204 and IRF7 were observed to co-localize in nucleus of SeV-induced *Irf3*^*−/−*^*Irf7*^*−/−*^ MEFs by immunofluorescent staining and confocal microscopy (Fig 4D). To reveal the function of this interaction, IFI204 or its 15 aa-deletion mutant IFI204 (IFI204-Δ225–239), which disrupts the interaction of IFI204 with IRF7 were co-transfected with IRF7 into *Irf3*^*−/−*^*Irf7*^*−/−*^MEFs. As shown in S9 Fig, IFI204 mutant (IFI204-Δ225–239) had significantly less inhibition than wildtype IFI204 to IRF7-mediated expression of IFNβ under the stimulation by SeV (S9 Fig). These data indicate that the interaction between IFI204 and IRF7 inhibits the type I IFN responses.

### IFI204 prevents IRF7 from binding with IRF7-specific promoter

**Fig 5:**
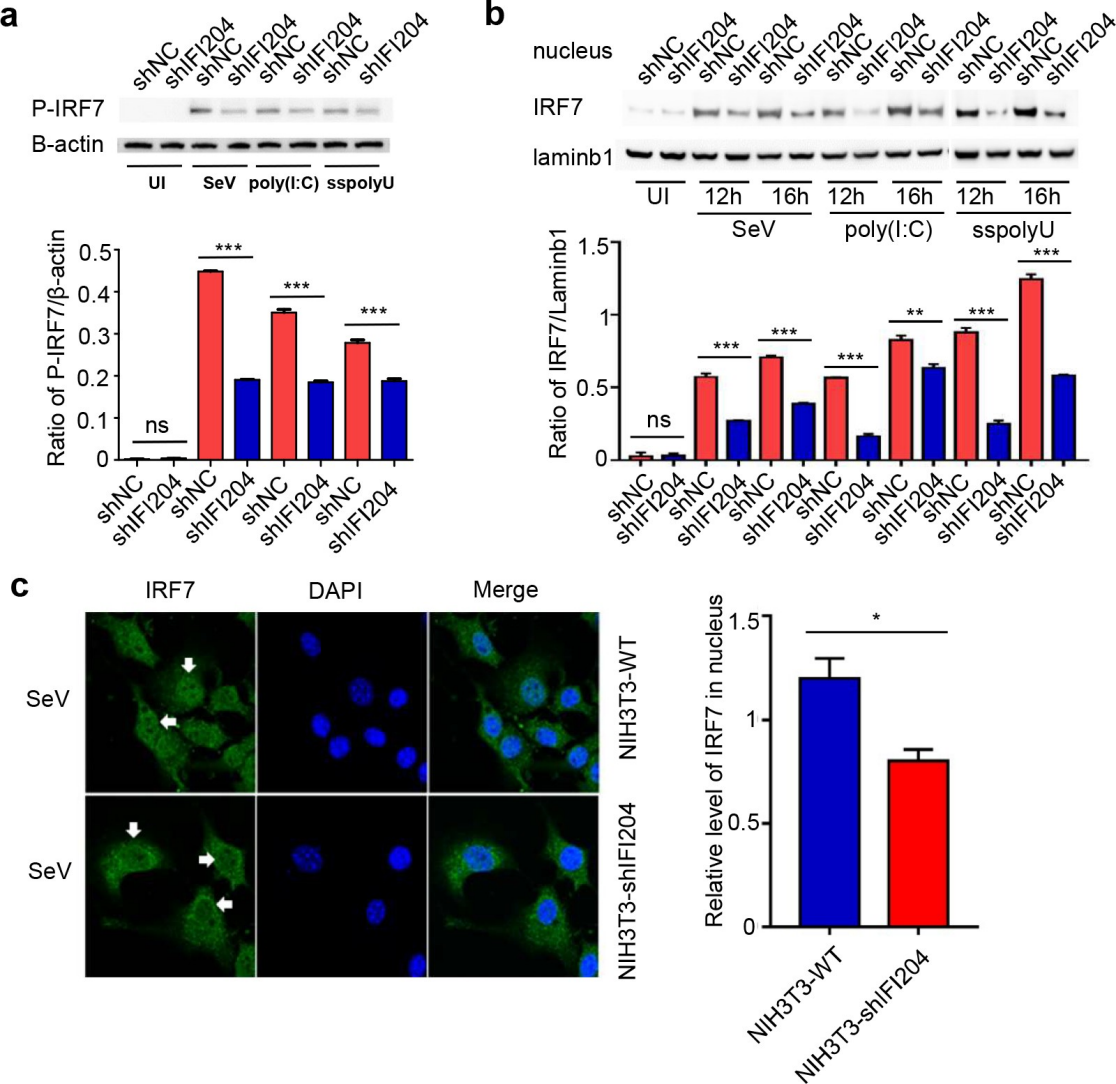
IFI204 promotes the nuclear retention of IRF7. (A) Immunoblot analysis of phosphorylation of IRF7 with rabbit mAb against phospho-IRF7-Ser437/438 (CST D6M2I) and β-actin in NIH3T3 cells infected by lentivirus-mediated shIFI204 or shNC as negative control. Cells were uninfected (UI) or infected with SeV or transfected with poly(I:C) or ssPolyU as indicated for 12 hrs. (B) Nucleo-cytoplasmic separation analysis of IRF7 in NIH3T3 cells infected by lentivirus-mediated shIFI204 or shNC as negative control. Cells were uninfected (UI) or infected with SeV or transfected with poly(I:C) or ssPolyU as indicated for 12 or 16 hrs. Nuclear lysates were tested by western blotting using anti-IRF7 and anti-lamin b1 antibodies. Levels of IRF7 was quantified by densitometry and normalized to lamin b1 protein levels. (C) Intracellular localization of endogenous IRF7 assessed by immunostaining followed by laser scanning confocal microscopy (left panel). NIH3T3 cells infected by lentivirus-mediated shIFI204 (NIH3T3-shIFI204) or shNC (NIH3T3-WT) were infected with SeV for 12 hrs. Then cells were washed with PBS and harvested for immunofluorescence analysis. Rabbit anti-IRF7 antibody was used as primary antibody and FITC-conjugated goat anti- rabbit IgG was used to detect IRF7 as shown in green fluorescence. DAPI (4, 6-diamidino-2-phenylindole) is used for nuclei staining as shown in blue fluorescence. The white arrow indicates the different protein levels of IRF7 in perinuclear region. Relative level of IRF7 in the nucleus was quantified by densitometry (right panel). *P < 0.05, **P < 0.01 and ***P < 0.001. Data are representative of two independent experiments (mean ± SD in A-C).

**Fig 6:**
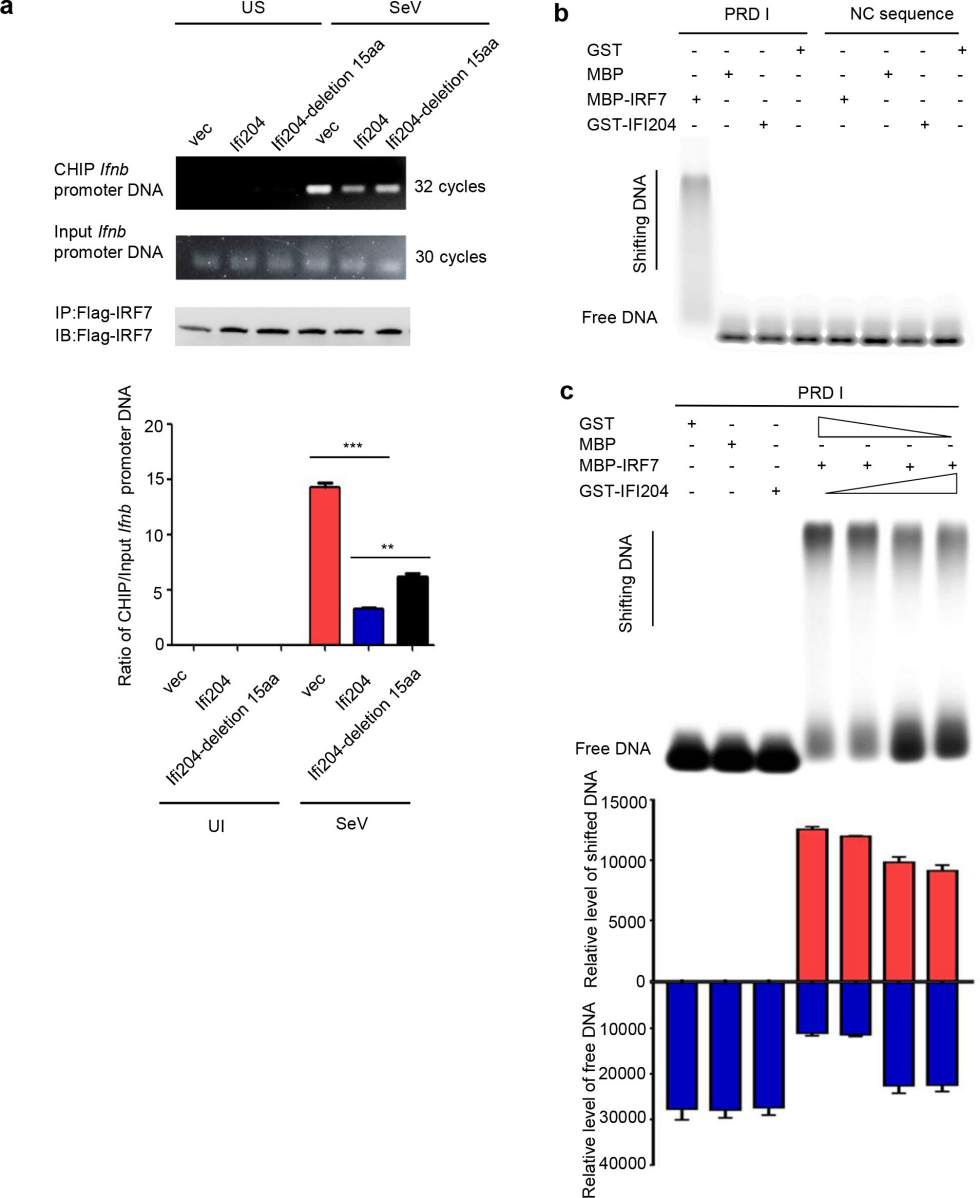
IFI204 inhibits IRF7 binding with its promoter. (A) CHIP analysis of the specific binding of IRF7 and its promoter DNA. IFI204 or IFI204 mutant was co-transfected with IRF7 into *Irf3*^*−/−*^*Irf7*^*−/−*^MEFs, which were unstimulated (US) or stimulated by SeV. Gel electrophoresis of the PCR products using Ifnb promoter primers and GAPDH primers (left panel). Levels of Ifnb promoter DNA were quantified by densitometry (right panel). (B and C) EMSA analysis of IRF7 binding with its promoter region. GST-IFI204, MBP-IRF7, GST and MBP were mixed with CY5-labeled PRD I or NC sequence probes as indicated. The NC sequence, GST and MBP are negative controls. Levels of shifted DNA and free DNA were quantified by densitometry (lower panel in C). **P < 0.01 and ***P < 0.001. Data are representative of two independent experiments.

Because the IFI204 and IRF7 were co-localized in the nucleus of RNA virus-infected cells, we hypothesized that IFI204 would hijack the imported IRF7, leading to suppression of the downstream transcription. The nucleo-cytoplasmic separation analysis revealed that knockdown of IFI204 could remarkably reduce the phosphorylation level of IRF7 and decrease the amount of nuclear IRF7 in SeV-, poly(I:C)- or ssPolyU-induced NIH3T3 cells (Fig 5A and 5B). Confocal fluorescence microscopy also confirmed this observation, indicating that IFI204 could interact with IRF7 and increase the amount of nuclear IRF7 (Fig 5C). These results suggest that negative regulation of IRF7-mediated IFN responses by IFI204 was not due to the inhibition of the phosphorylation and nuclear import of IRF7, but due to blocking a downstream step, such as DNA-binding. Therefore, we adopted the CHIP and EMSA analysis to investigate the binding efficiency of IRF7 and its cognate promoter DNA. As shown in Fig 6, the CHIP analysis demonstrated that IFI204 remarkably inhibits the binding of IRF7 and its promoter DNA. In contrast, the IFI204 mutant (IFI204-Δ225–239) had significantly less inhibition on the binding of IRF7 with its specific promoter DNA (Fig 6A). The EMSA analysis showed that the IRF7 could bind to PRDI region on the promoter of IFN-β gene as previously reported [36], while IFI204 could not bind with the IRF7-specific promoter (Fig 6B). This result excludes the possibility that IFI204 directly binds to PRDI promoter to inhibit its transcription. As shown in Fig 6C, the binding of IRF7 and PRDI was remarkably attenuated by the IFI204 in a dose-dependent manner (Fig 6C). Together, the results indicate that the interaction of IFI204 and IRF7 prevents IRF7 from binding to IRF7-specific promoter DNA.

### P200 family proteins possess conserved function in the inhibition of IRF7-mediated type I IFNs responses

**Fig 7:**
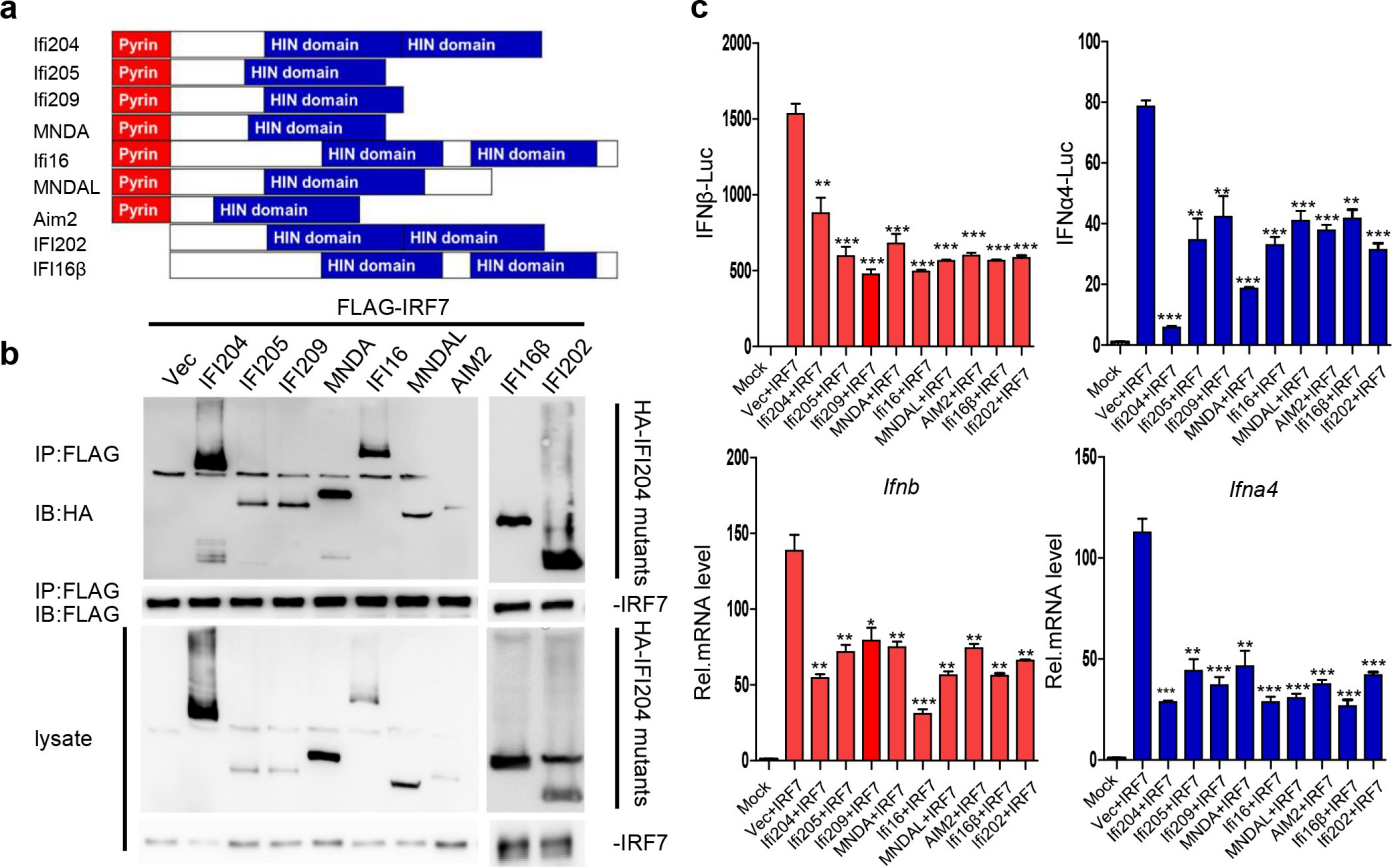
Conserved function of p200 family proteins in the inhibition of type I IFNs responses. (A) Schematic diagram of the domain structure of p200 family proteins. (B) Co-immunoprecipitation (IP, with anti-Flag) and immunoblot (IB, with anti-HA) analysis of HEK293T cells that were transfected with plasmids encoding Flag-IRF7 and HA-(IFI204, IFI205, IFI209, MNDA, IFI16, MNDAL, AIM2, IFI202 and IFI16β) for 24 hrs. Cell lysate was analyzed by immunoblot with anti-Flag and anti-HA antibodies. (C) Dual luciferase assays for analyzing the promoter activity of IFNβ or IFNα4 in *Irf3*^*−/−*^*Irf7*^*−/−*^MEFs co-transfected with IFNβ or IFNα4 promoter luciferase reporter plasmids and the indicated plasmids, respectively. The Renilla expression plasmid (pRL-TK, 10 ng) was co-transfected as an internal control (upper graphs). qRT-PCR analysis of *Ifnb* or *Ifna4* in *Irf3*^*−/−*^*Irf7*^*−/−*^ MEFs transfected with the indicated plasmids (lower graphs). 24 hrs post-transfection, cells were treated with SeV for 12 hrs. *P < 0.05, **P < 0.01 and ***P < 0.001. Data are representative of three independent experiments (mean ± SD in C).

**Fig 8:**
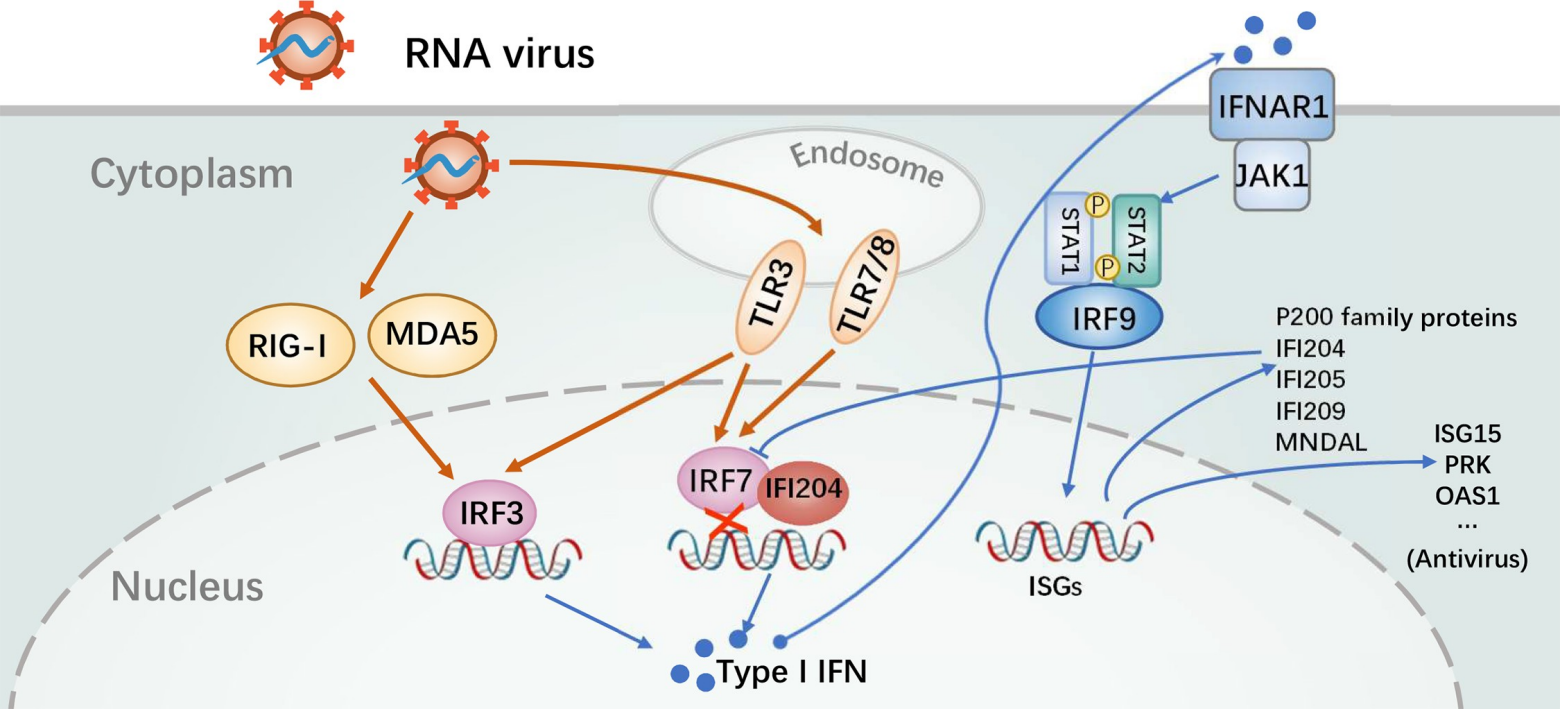
Proposed molecular mechanism for the function of p200 family proteins (IFI204) to inhibit IRF7-mediated activation of type I IFNs responses. Schematic diagram demonstrates the mechanism of p200 family proteins to suppress type I IFNs responses. Late after RNA virus infection, the highly expressed p200 family proteins, such as IFI204, interact with IRF7 in the nucleus and such interactions lead to the negative regulation of type I IFNs responses.

The HIN domain is highly conserved among p200 family proteins (Fig 7A). Therefore, we investigated whether other proteins of the p200 family possess the similar function as that of IFI204. As shown in Fig 7B and 7C, the representative p200 family proteins, including IFI205, IFI209, IFI202 and MNDAL from mice and MNDA, IFI16, IFI16β and AIM2 from human, all could interact with IRF7 (Fig 7B) and showed a significant repression of type I IFNs response in SeV-induced IRF7-transfected *Irf3*^*−/−*^*Irf7*^*−/−*^ MEFs (Fig 7C). To confirm this result, IFI205 was overexpressed in BMDCs and the expression level of IFNs were repressed (S10A Fig). In contrast, knockdown of IFI205 stimulated SeV-, or poly(I:C)-induced expressions of IFNs in NIH3T3 cells (S10B Fig). Taken together, p200 family proteins, which contain the conserved HIN domain might play a negative regulation role in IRF7-mediated type I IFN signaling pathway. During RNA virus infection, the activated IRF7 would be bound with HIN domain of p200 family proteins, such as IFI204, IFI205 and IFI16, thus inhibiting the subsequent gene transcription (Fig 8). In contrast, HSV-1 infection could not effectively activate the IRF7-mediated signaling pathway but promote cGAS-STING DNA sensing pathway to facilitate IRF3 activation.

## Discussion

IFI204/IFI16, known as ALRs and expressed constitutively in host cells, are involved in the sensing of intracellular DNA derived from pathogens and subsequent induction of the production of type I IFNs as well as proinflammatory mediators via STING-IRF3 pathway [19, 24]. However, Gray and colleagues demonstrated that ALRs, including IFI204, are dispensable for the interferon response to intracellular DNA in mice with knockout of all PYHIN proteins [33]. Two later studies reported that IFI16 cooperates with cGAS-STING pathway to promote the type I IFNs during the DNA virus infection [28, 29]. In this study, we showed that IFI204 could promote the expression of IFNβ via IRF3-mediated signaling pathway (S5 Fig and Fig 3) but down regulate the IRF7-mediated IFN response. Why inconsistent results were obtained from different studies is still one unanswered question. We propose that PYHIN proteins may not be essential in DNA sensing (as shown by Gray et al.) but involved in the regulation of interferon responses (as shown in this study and by different laboratories). The regulatory function of p200 family members may involve more than one PYHIN protein, and when all PYHIN proteins are knocked out, such regulation could not be observed and, instead, the regulation of interferon response is compensated by other regulation system. Another possible explanation for the discrepancy may be due to the use of different inducers (or viruses) and cell types. More studies are needed to clarify these issues in the future but they are already beyond the range of current work, which focuses to reveal the negative regulatory function of p200 family proteins in IRF7-mediated type I IFN responses (Figs 2, 3 and 7).

P200 family proteins are IFN-inducible proteins, belonging to the category of ISGs [37]. Many ISGs were reported to positively regulate the IFN responses. However, only a few ISGs are known to negatively regulate type I IFN responses and proinflammatory mediators. Here, we demonstrated that the IFI204 and several other p200 family proteins were highly induced in both DNA and RNA virus infection (Fig 1 and S2 Fig). Intriguingly, IFI204 specifically inhibits the IRF7-mediated type I IFNs response in RNA virus infection (Figs 2, 3 and 7). The infection of DNA virus HSV-1 could not effectively stimulate the IRF7-mediated signaling pathway as shown in Fig 3 and in previous studies [19, 24]. However, in the absence of IRF3, IFI204 could still inhibit type I IFN responses initiated by the self-activating IRF7-Δ247–467 mutant in the context of HSV-1 infection (Fig 3B and 3A). Such results suggest a specific function of IFI204 in suppression of IRF7-mediated type I IFNs responses in whatever way IRF7 is activated.

Negative regulation of IRF7 could take place at the stage of IRF phosphorylation, nuclear translocation and binding with the promoter. Our results showed that IFI204 did not inhibit the phosphorylation and nuclear import of IRF7 but increase the phosphorylation level and nuclear retention of IRF7 to some degree (Fig 5), suggesting that inhibitory function of IFI204 is not due to the suppression of IRF7 activation. By CHIP and EMSA assays, we revealed that IFI204 could specifically interact with the DNA binding domain (DBD) of IRF7 and consequently inhibit its subsequent binding with promoter DNA (Fig 6). IFI204 itself does not have specific binding with IRF7 promoter (Fig 6), indicating that IFI204 does not directly compete with IRF7 in the DNA binding but prevent IRF7 from promoter binding via its interaction with IRF7 DBD.

The type I IFN response is critical for viral clearance. However, the whole signaling pathway should be tightly regulated to avoid overproduction of IFNs and inflammatory factors that can cause tissue damage and even autoimmune diseases [38]. For example, the acute coronaviral infection such as Middle East respiratory syndrome coronavirus (MERS-CoV) and severe acute respiratory syndrome coronavirus (SARS-CoV), and overdosed IFNα in antiviral treatment can cause tissue damage due to the hyper-inflammatory responses [39]. Here we use MHV that belongs to the same genus *betacoronavirus* as SARS-CoV and MERS-CoV to mimic the acute RNA virus infection. In our MHV-infected BMDC model, both IRF3- and IRF7-mediated pathways of type I IFNs and some subsequent proinflammatory mediators were stimulated (Figs 1 and 3, S1 and S6 Figs). Notably, IRF7-mediated pathway of type I IFN responses is mainly initiated by ssRNA sensor TLR7, and previous studies reported that the over-expression or activation of TLR7 is associated with autoimmune diseases [40–42]. The over-expression and activation of TLR7 as well as the stimulation of IFNα were also observed in our MHV-infection BMDCs model (Figs 2 and 3, S6 Fig), which is consistent with clinical symptoms. Our current study revealed a novel negative regulation mechanism in the RNA virus-induced IRF7-mediated IFN responses.

The regulation of type I IFN signaling pathway is dynamic sequential processes [43]. Taken together the results of this study and previous knowledge, we propose a model for the function of IFI204 in negative regulation of IFN responses as shown in Fig 8. Many host negative regulators of IFN responses are constitutively expressed in cells [43]. In contrast, p200 family protein IFI204/IFI16 is up-regulated during virus infection (Fig 1). We may assume that induced expression of IFI204/IFI16 might exert more robust regulation of IFN responses.

In summary, our current work revealed a previously unknown negative regulation mechanism of IRF7-mediated IFN responses by PYHIN proteins including IFI204/IFI16. Such knowledge may help to better understand the accurate regulation system of IFN signaling pathway during viral infection.

## Materials and methods

### Ethics statement

All mice were housed in the specific pathogen-free animal facility at Wuhan University and all animal experiments were in accordance with protocols that were adhered to the Chinese National Laboratory Animal-Guideline for Ethical Review of Animal Welfare and approved by the Animal Care and Use Committee of Wuhan University (NO.15060C). The mice were euthanatized with CO_2_ followed by various studies.

### Cells, virus and reagents

HEK293T, NIH3T3, RAW264.7 and A549 cells were obtained from American Tissue Culture Collection (ATCC). *Irf3*^*−/−*^*Irf7*^*−/−*^ MEFs were kindly provided by Dr. Pinghui Feng (University of Southern California). Sendai virus (SeV) and vesicular stomatitis virus (VSV) were kindly provided by Dr. Hong-Bing Shu (Wuhan University). Herpes simplex virus-1 (HSV-1) were kindly provided by Dr. Yu Liu (Wuhan University). Murine hepatitis virus (MHV) strain A59, which is positive single-stranded RNA (+ssRNA) virus and belongs to the *beta coronavirus* in *Nidovirales* that includes Middle East respiratory syndrome coronavirus (MERS-CoV) and severe acute respiratory syndrome coronavirus (SARS-CoV), was used as described previously [44, 45]. The chemical reagents used in this study were from the following manufacturers: poly(I:C) (Invivogen); ssPolyU (Invivogen); One Step Seamless Cloning kit (Aidlab,China); GM-CSF (Peprotech); TRIzol (Invitrogen).

### Plasmids

The complementary DNA (cDNA) of *Ifi204*, *Ifi205*, *Ifi209*, *Mndal* and *Irf7* were amplified from total RNA obtained from MHV-stimulated BMDCs, respectively. *IFI16*, *MNDA* and *AIM2* complementary DNA (cDNA) were amplified from cDNA library obtained from Dr. Jia-Huai Han (Xiamen University). *Irf7* was cloned into pRK and pMAL-CX2 by recombinase-mediated recombination. To generate the self-activating IRF7 mutant IRF7-Δ247–467 plasmid (pRK-Flag-IRF7Δ247–467), the plasmid pRK-Flag-IRF7 was amplified by PCR to delete the nucleotides 739 to 1401 of IRF7. The PCR product was digested with Dpn1 (Thermo Fisher Scientific) to remove template plasmids and then ligated by recombinase-mediated recombination. The other genes and the mutants of *Ifi204* or *Irf7* were cloned into pCAGGS or pGEX-4T-1, respectively as mentioned. All the plasmids were confirmed by DNA sequencing. The PCR primers are listed in S1 Table.

### Preparation of BMDCs

The BMDCs were prepared as previously described [46]. Bone marrow cells were isolated from mouse tibia and femur. The cells were cultured for 7–9 days in medium containing mouse cytokine GM-CSF (20 ng/ml) to induce BMDCs. The positive rate is 85% by the detection of flow cytometry.

### siRNA and gRNA

The sequences of siRNA and gRNA are as following.

siIFI204-1: CCCAUUUGAAUAUGAAUCATT

siIFI204-2: GUGCCCAACAGUAUUAUCATT

siIFI204-3: GCAGUAAACUAAGACUUGUTT

siIFI205-1: CCACAUGCCAGUCACCAAUTT

si-m-STING: ATGATTCTACTATCGTCTTA

si-h-STING: GCATGGTCATATTACATCGG

si-m-CGAS: CCAGGATTGAGCTACAAGAAT

si-h-CGAS: GCAACTACGACTAAAGCCATTT

gRNA (*Ifi204*): TATACCAACGCTTGAAGACC

### Lentiviral packaging and infection

The experiments were performed as previously described [47]. HEK293T cells (in 100-mm dish) were co-transfected with 2 μg LentiV2, 1.5 μg psPAX2 and 1.5 μg pMD2.G. Cell supernatants were collected at 48 hrs after transfection and the lentivirus in cell supernatants was conserved at -80°C after high-speed centrifugation and filtration. Supernatants were used to infect NIH3T3 cells and A549 cells. Cells were infected twice to obtain higher transduction efficiency, and puromycin (NIH3T3 3 μg/ml, A549 1.5 μg/ml) was used to screen the positive cells.

### Western blotting

Cells were washed once with PBS and lysed in lysis buffer (50mM Tris, pH 7.6, 1% Triton X-100 and 150 mM NaCl). Protein samples were mixed with SDS loading buffer and boiled for 5 mins. Samples were resolved by SDS–PAGE, transferred to nitrocellulose membrane (GE Healthcare), blocked with TBS containing 0.1% Tween-20 with 5% skim milk and probed with antibodies to IFI204 (Abcam), IFI16 (Abclonal), IRF7 (Abcam), Phospho-IRF-7 (CST D6M2I that detects Ser437 and Ser438 phsphorylation), Flag (Sigma), HA (Roche), GST (Proteintech), MBP (Abclonal), LaminB1 (Proteintech), β-actin (Abclonal) and IgG (Santa Cruz).

### RNA isolation and quantitative RT-PCR

Total RNA was isolated from cells with TRIzol reagent under the instruction of the manufacturer. The mRNAs were reverse transcribed into cDNA by PrimeScript RT reagent Kit (Takara). The cDNA was amplified by a fast two-step amplification program using SYBR Green Fast qPCR MasterMix (Cat No. 11201–11203*; Yeasen, China). GAPDH was used to normalize the input samples via the ΔΔCt method. The RT-PCR primers are listed in S2 Table.

### ELISA

Cytokines were detected by using mouse IFNα ELISA kit (R&D) under the instruction of manufacturer.

### Purification of recombinant IFI204 and IRF7

GST-IFI204 and MBP-IRF7 were expressed in *Escherichia coli* BL21 (Invitrogen) at 16°C. The cells were harvested after a 12 hrs induction period in the presence of 0.5 mM isopropyl-β-D-thiogalactopyranoside (IPTG). Cells were lysed by enzymatic lysis buffer (50 mM Tris-HCl [pH 7.6], 150 mM NaCl, 1 mM EDTA, 1 mM dithiothreitol [DTT], 0.1 mg/ml lysozyme, 0.05% NP-40). Proteins were purified using GST-Fast Flow according to the manufacturer's recommendation. Purified proteins were quantified and stored in PBS (pH 7.4) at -80°C. MBP-tagged fusion proteins were purified from cell lysates by affinity chromatography using MBP-Tag Dextrin Resin (NEB) according to the instruction of the manufacturer.

### Nuclear/cytoplasmic fractionation

Nuclear and cytoplasmic extracts were prepared with nuclear cytoplasmic extraction kit (Applygen Technologies) according to the manufacturer's manual.

### EMSA

The purified target proteins were mixed in binding buffer (400 mM Tris-HCl [pH 8.0], 50 mM DTT) and incubated on ice for 30 mins. The CY5-labeled double-stranded oligonucleotides of the IFNβ promoter (PRDI region) (5’-GAGAAGTGAAAGTGGAGAAGTGAAAGTGGAGAAGTGAAAGTGGAGAAGTGAAAGTG-3’) and negative control sequence (NC-sequence) (5’-CATGGGTGGCCACAACTTCGCCTTTTCGTCAGTAATACACGATTGGAGAAAGTTAC-3’) were add to the reaction mix and incubated at room temperature for 30 mins. The samples were separated in 1% agarose gel at 130V for 1 h and subjected to the analysis with Typhoon FLA9500 (GE).

### Chromatin immunoprecipitation (ChIP)

Chromatin immunoprecipitation was carried out using a ChIP Assay kit (Beyotime, catalog number P2078) according to manufacturer's instructions. Briefly, IFI204 or IFI204 mutant were co-transfected with IRF7 into *Irf3*^*−/−*^*Irf7*^*−/−*^ MEFs, which were stimulated by SeV. 8 hrs post infection, cells were fixed using 1% formaldehyde for 10 min at 37°C followed by neutralization using 125 mm glycine. The cells were then lysed on ice in SDS lysis buffer (50 mm Tris-HCl, pH8.0, 10 mm EDTA, 150 mm NaCl, 1% SDS) supplemented with 1× proteinase inhibitor mixture (Roche Applied Science). Then the lysate was sonicated on ice to break the genome into 200–1000 bp in size. We used ANTI-FLAG M2 Affinity Gel (Product No. A2220) to allow precipitation of FLAG-IRF7-associated DNA fragments by incubation at 4°C overnight followed by washing with different washing buffers as follows: low salt immune complex wash buffer, one time; high salt immune complex wash buffer, one time; LiCl immune complex wash buffer, one time; and Tris-EDTA buffer, two times. The DNA components in the precipitation were finally extracted using a standard phenol-chloroform method. PCR amplifications were conducted using the IFNβ promoter-specific primers as belows:

IFNβ promoter-forward: 5′- AGCCAGGAGCTTGAATAAAA -3′

IFNβ promoter-reverse: 5′-GATCTTCCTTCATGGCCTGT-3′

GAPDH-forward: 5′- CGACTTCAACAGCAACTCCCACTCTTCC -3′

GAPDH-reverse: 5′- TGGGTGGTCCAGGGTTTCTTACTCCTT -3′

### Transcriptome and proteome analysis

RNA-Seq data processing:

The generation and sequencing of cDNA libraries were done on Illumina Hiseq-2500 platform to generate 150bp PE reads. Raw RNA-seq reads were trimmed using cutadapt (v1.13) of adaptor sequences AGATCGGAAGAGCACACGTCTGAACTCCAG, AGATCGGAAGAGCGTCGTGTAGGGAAAGAG, and mapped to Mouse Genome (mm10) using STAR (v2.5.3a) with GENCODE (vM18) gene annotations. The number of reads mapping to each gene were calculated using HTSeq (v0.11.2). Differential gene transcriptions were analyzed using DESeq2 (v1.18.1) with log2 (Fold Change) > 3.

Proteome data processing:

The samples were combined and analyzed in triplicates by EASY-nLC 1000 system (Thermo Scientific, Odense, Denmark) and Q Exactive HF mass spectrometer system (Thermo Scientific, San Jose, CA, USA). The preliminary mass spectrum data were analyzed by MaxQuant software suite (v1.5.0.25). The downstream data processing and bioinformatics analysis are carried out by Perseus software (version 1.4.2.30). Differentially expressed proteins were analyzed using Perseus software (version 1.4.2.30) with (Fold Change) > 1.5.

### Statistical analysis

Data were analyzed using GraphPad Prism 5 software (GraphPad, La Jolla, CA, USA).

*P < 0.05, **P < 0.01 and ***P < 0.001 denote significant differences.

## Supporting information

S1 Fig Various interferons and inflammatory factors were highly expressed in MHV-infected BMDCs. Transcriptome analysis to measure the relative expression levels of *Ifnb*, *Ifna1*, *Ifna4*, *Ifna6*, *Il6*, *Tnf* and *Nfkb* in BMDCs with or without MHV-infection (mock). BMDCs were infected by MHV-A59 and collected at 18 hpi. Mean ± SEM represents the average value of two independent experiments.
(TIF)

S2 Fig IFI204 is highly induced by Type I Interferons in HSV-1 infected BMDCs. (A and B) qRT-PCR analysis of Ifi204 in HSV-1-infected BMDCs (A) and *Ifnar*^-/-^ BMDCs (B) at different time points as indicated.
(TIF)

S3 Fig Knock-down or knockout of IFI204 or IFI16 in various cells. (A) qRT-PCR analysis to evaluate the efficiency of siRNAs. NIH3T3 cells were transfected with negative control siRNA (si-NC) or *Ifi204*-targeting siRNAs (si-IFI204-1, si-IFI204-2 and si-IFI204-3). The cells were collected 24 hrs post transfection and subjected to qRT-PCR. (B) Immunoblot analysis to evaluate the efficiency of siRNAs. HEK293T cells were co-transfected with plasmids encoding HA-tagged IFI204 and either si-NC as a control or *Ifi204*-targeting siRNAs (si-IFI204-1, si-IFI204-2 and si-IFI204-3). The cells were collected at 36 hrs post transfection and subjected to western blotting with anti-HA or anti-β-actin antibodies. (C) qRT-PCR analysis of *Ifi204* in NIH3T3 cells stably expressing shIFI204. The NIH3T3 cells were infected by lentivirus-mediated small hairpin RNA targeting *Ifi204* (shIfi204) to generate stable IFI204 knockdown cells. The shNC is negative control. (D) qRT-PCR analysis of the mRNA level of Ifi204 in BMDCs stably expressing shIFI204. The BMDCs were infected by lentivirus-shIfi204 to generate stable IFI204 knockdown BMDCs. The shNC is negative control. (E) Immunoblot analysis (with anti-IFI16 or anti-β-actin) of *IFI16*^+/+^ A549 and *IFI16*^-/-^ A549. (F) Sequence analysis of *IFI16*^+/+^ A549 and *IFI16*^-/-^ A549. The open reading frame of IFI16 is changed by the insertion of an additional T as indicated by red arrow. **P < 0.01 and ***P < 0.001. Data are representative of three independent experiments (mean ± SD in A, C and D).
(TIF)

S4 Fig IFI204 inhibits the production of type I IFNs in RAW264.7 cell lines. (A) qRT-PCR analysis of *Ifi204* in stable shIFI204 RAW264.7 cells. The RAW264.7 cells were infected by lentivirus-shIfi204 to generate stable IFI204 knockdown cells. The shNC is negative control. (B) qRT-PCR analysis of *Ifnb*, *Ifna1* and *Ifna4* in stable shNC or shIFI204 RAW264.7 cells. The cells were stimulated by the infection of VSV for 8 hrs. The unstimulated cells (US) are controls. **P < 0.01 and ***P < 0.001. Data are representative of three independent experiments (mean ± SD in A and B).
(TIF)

S5 Fig IFI204 activates the production of type I IFNs during the infection of DNA virus. (A) qRT-PCR analysis of *Ifnb* in stable IFI204-knockdown NIH3T3 cells transfected with si-*Sting* or si-*Cgas*. The shNC is control. The cells were unstimulated (US) or stimulated with HSV-1 (MOI = 1) as indicated for 12 hrs. (B) qRT-PCR analysis of *IFNβ* in *IFI16*^-/-^ A549 cells transfected with si-CGAS or si-STING. The *IFI16*^+/+^ A549 cells is control. The cells were unstimulated (US) or stimulated with HSV-1 (MOI = 1) as indicated for 12 hrs. **P < 0.01 and ***P < 0.001. Data are representative of three independent experiments (mean ± SD in A and B).
(TIF)

S6 Fig Both IRF3- and IRF7-mediated signaling pathway were stimulated in MHV-infected BMDCs. Transcriptome analysis to measure the relative expression levels of *Rig-I*, *Mda5*, *Tbk1*, *Traf3*, *Traf6*, *Tlr3*, *Tlr7* and *Irf7*. BMDCs were infected by MHV and collected at 18 hpi. Data are representative of two independent experiments (mean ± SD).
(TIF)

S7 Fig IFI204 interacts with IRF7. Co-immunoprecipitation and immunoblot analysis of IFI204 and IRF3 (A) or IRF7 (B and C). (A and B) HEK293T cells were co-transfected with plasmids encoding HA-IFI204 or its vector (vec) as control and Flag-IRF3 (A) or Flag-IRF7 (B) as indicated. Cell lysate was immunoprecipitated with anti-Flag and analyzed by immunoblot with anti-Flag or anti-HA antibodies. (C) NIH3T3 cells were infected with SeV for 8 hrs. Cell lysate was immunoprecipitated with anti-IRF7 antibody or control IgG, and analyzed by immunoblotting with anti-IRF7 or anti-IFI204 antibodies to detect the association of endogenous proteins.
(TIF)

S8 Fig IFI204 interacts with DBD domain of IRF7. Co-immunoprecipitation and immunoblot analysis of HEK293T cells, which were transfected with plasmids encoding HA-IFI204 and Flag-IRF7 or its mutants for 24 hrs. Cell lysate was analyzed by immunoblot with anti-Flag and anti-HA antibodies. Schematic diagram to show the regions of IRF7 interacting with IFI204 (right panel).
(TIF)

S9 Fig IFI204 mutant reverts the inhibition of type I IFNs response. (A) Dual luciferase assays for analyzing the promoter activity of IFNβ in *Irf3*^*−/−*^*Irf7*^*−/−*^ MEFs co-transfected with 200 ng IFNβ promoter luciferase reporter plasmid, 250 ng IRF7, IFI204 or IFI204 mutant as indicated. The Renilla expression plasmid (pRL-TK, 10 ng) was co-transfected as an internal control. 24 hrs post-transfection, cells were unstimulated (US) or stimulated with SeV for 12 hrs. (B) qRT-PCR analysis of *Ifnb* in *Irf3*^*−/−*^*Irf7*^*−/−*^ MEFs co-transfected with IRF7 and IFI204 or IFI204 mutant as indicated. 24 hrs post-transfection, cells were unstimulated (US) or stimulated with SeV for 12 hrs. **P < 0.01 and ***P < 0.001. Data are representative of three independent experiments (mean ± SD in A and B).
(TIF)

S10 Fig IFI205 inhibits the type I IFNs responses. (A) qRT-PCR analysis of *Ifnb*, *Ifna4*, *Ifit1* and ELISA analysis of IFNα in BMDCs stably expressing IFI205 or its vector (Vec) as negative control. BMDCs were infected by lentivirus-IFI205 or lentivirus-vector (Vec), respectively. The cells were unstimulated (US) or stimulated by SeV for 8 hrs as indicated. (B) qRT-PCR analysis of *Ifnb*, *Ifna4*, *Ifit1* and ELISA analysis of IFNα in NIH3T3 cells transfected with si-*Ifi205* and siNC as negative control. The cells were unstimulated (US) or stimulated by SeV or poly(I:C) for 8 hrs as indicated. *P < 0.05, **P < 0.01 and ***P < 0.001. Data are representative of three independent experiments (mean ± SD in A and B).
(TIF)

S1 Table Primers for PCR. (DOCX)

S2 Table Primers for mRNA quantification. (DOCX)
