# Supplementary figures and images for: P200 family protein IFI204 negatively regulates type I interferon responses by targeting IRF7 in nucleus
Source: PLoS Pathog. 2019 Oct 11;15(10):e1008079. doi: 10.1371/journal.ppat.1008079 (PMC6818788; doi:10.1371/journal.ppat.1008079)

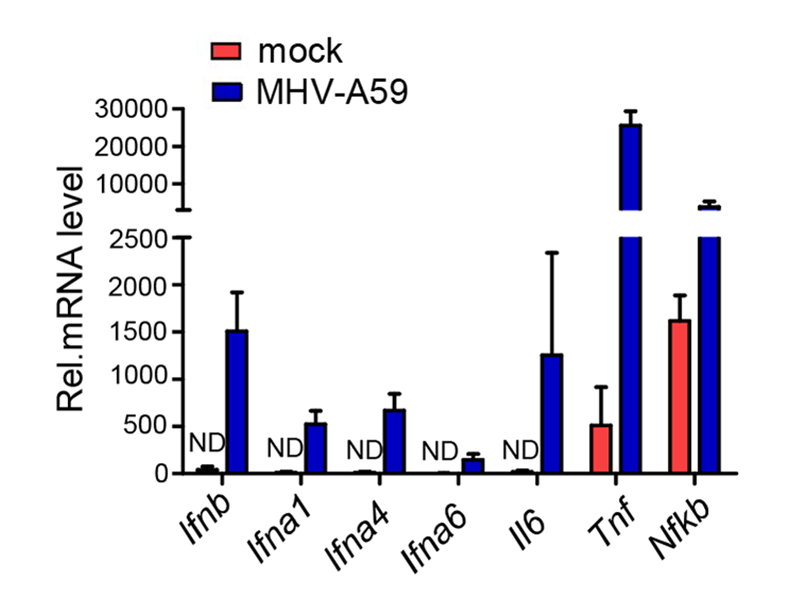

Supplement: S1 Fig — Transcriptome analysis to measure the relative expression levels of Ifnb, Ifna1, Ifna4, Ifna6, Il6, Tnf and Nfkb in BMDCs with or without MHV-infection (mock). BMDCs were infected by MHV-A59 and collected at 18 hpi. Mean ± SEM represents the average value of two independent experiments. (TIF) [file ppat.1008079.s001.tif]

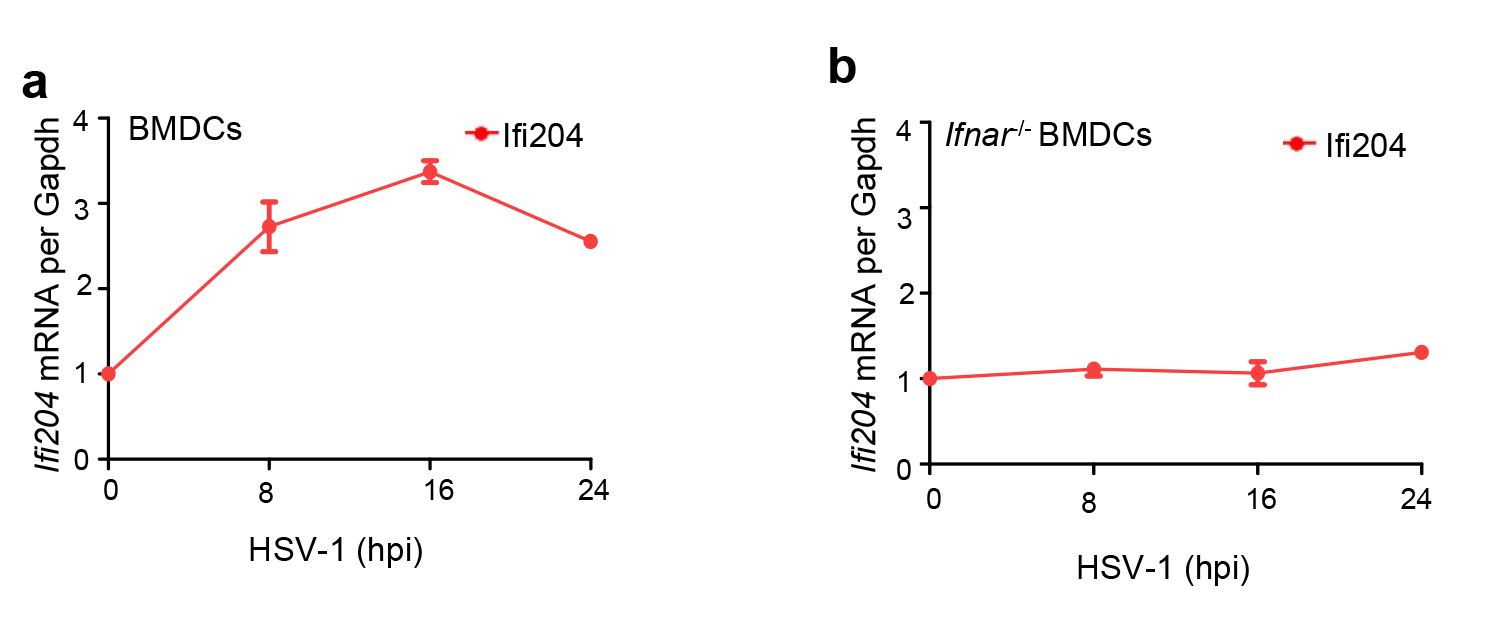

Supplement: S2 Fig — (A and B) qRT-PCR analysis of Ifi204 in HSV-1-infected BMDCs (A) and Ifnar-/- BMDCs (B) at different time points as indicated. (TIF) [file ppat.1008079.s002.tif]

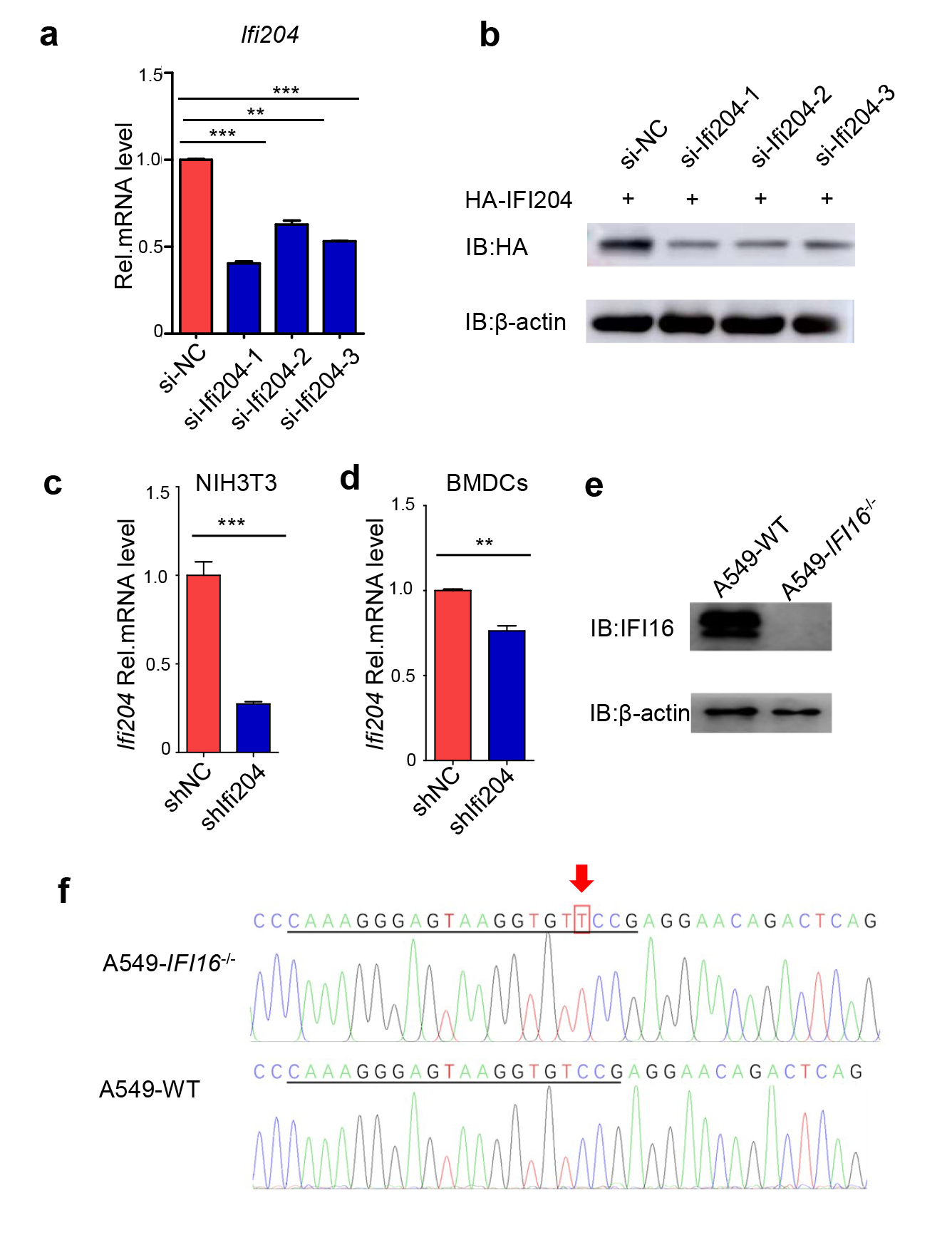

Supplement: S3 Fig — (A) qRT-PCR analysis to evaluate the efficiency of siRNAs. NIH3T3 cells were transfected with negative control siRNA (si-NC) or Ifi204-targeting siRNAs (si-IFI204-1, si-IFI204-2 and si-IFI204-3). The cells were collected 24 hrs post transfection and subjected to qRT-PCR. (B) Immunoblot analysis to evaluate the efficiency of siRNAs. HEK293T cells were co-transfected with plasmids encoding HA-tagged IFI204 and either si-NC as a control or Ifi204-targeting siRNAs (si-IFI204-1, si-IFI204-2 and si-IFI204-3). The cells were collected at 36 hrs post transfection and subjected to western blotting with anti-HA or anti-β-actin antibodies. (C) qRT-PCR analysis of Ifi204 in NIH3T3 cells stably expressing shIFI204. The NIH3T3 cells were infected by lentivirus-mediated small hairpin RNA targeting Ifi204 (shIfi204) to generate stable IFI204 knockdown cells. The shNC is negative control. (D) qRT-PCR analysis of the mRNA level of Ifi204 in BMDCs stably expressing shIFI204. The BMDCs were infected by lentivirus-shIfi204 to generate stable IFI204 knockdown BMDCs. The shNC is negative control. (E) Immunoblot analysis (with anti-IFI16 or anti-β-actin) of IFI16+/+ A549 and IFI16-/- A549. (F) Sequence analysis of IFI16+/+ A549 and IFI16-/- A549. The open reading frame of IFI16 is changed by the insertion of an additional T as indicated by red arrow. **P < 0.01 and ***P < 0.001. Data are representative of three independent experiments (mean ± SD in A, C and D). (TIF) [file ppat.1008079.s003.tif]

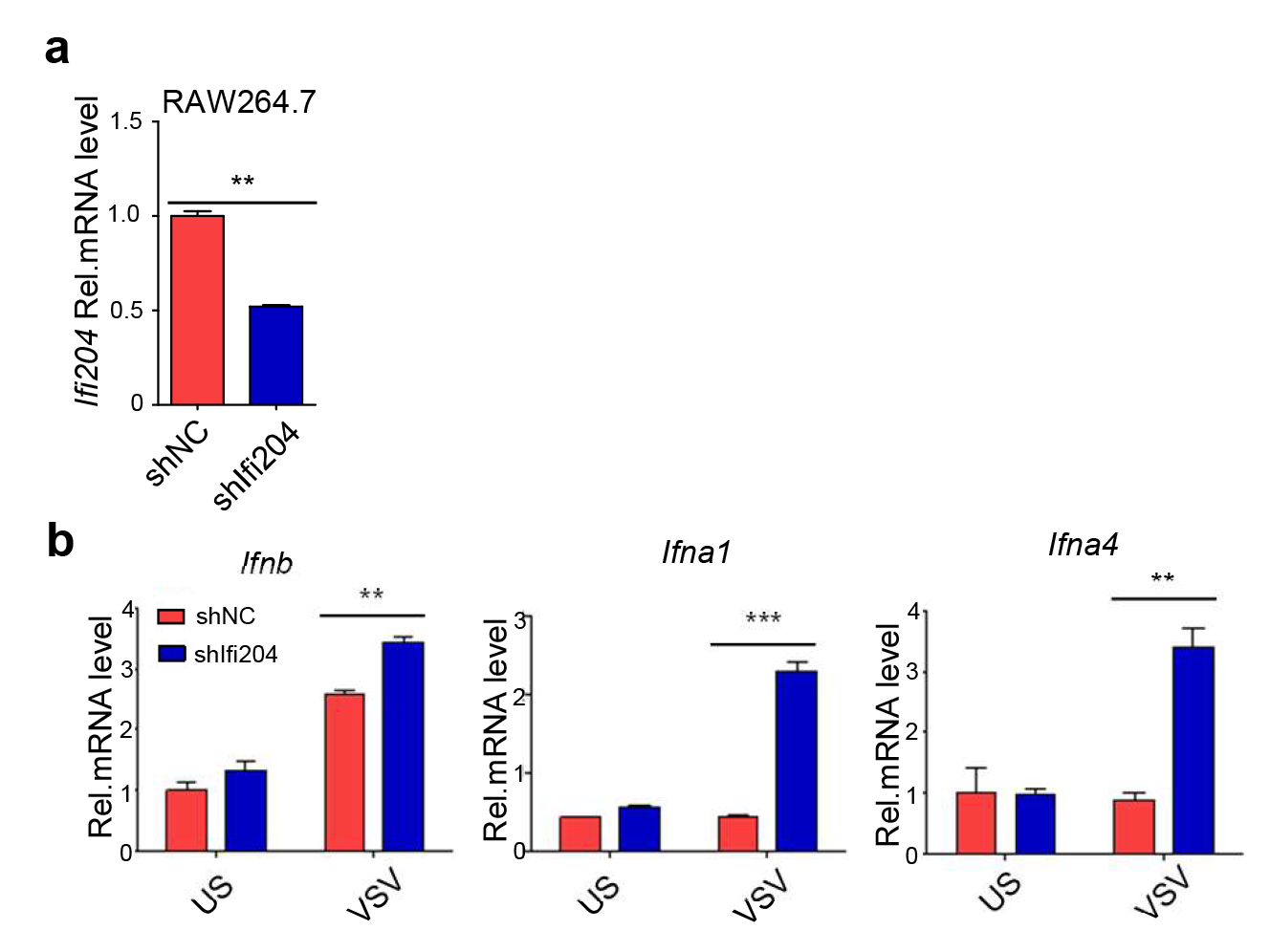

Supplement: S4 Fig — (A) qRT-PCR analysis of Ifi204 in stable shIFI204 RAW264.7 cells. The RAW264.7 cells were infected by lentivirus-shIfi204 to generate stable IFI204 knockdown cells. The shNC is negative control. (B) qRT-PCR analysis of Ifnb, Ifna1 and Ifna4 in stable shNC or shIFI204 RAW264.7 cells. The cells were stimulated by the infection of VSV for 8 hrs. The unstimulated cells (US) are controls. **P < 0.01 and ***P < 0.001. Data are representative of three independent experiments (mean ± SD in A and B). (TIF) [file ppat.1008079.s004.tif]

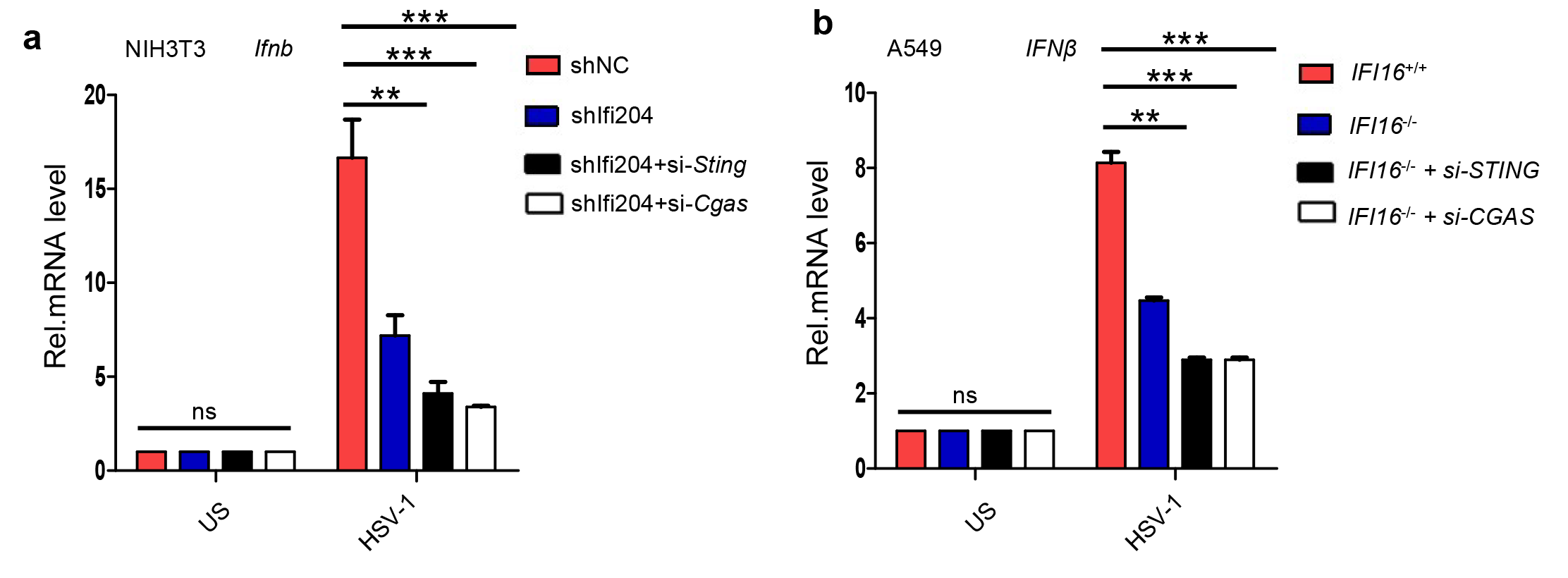

Supplement: S5 Fig — (A) qRT-PCR analysis of Ifnb in stable IFI204-knockdown NIH3T3 cells transfected with si-Sting or si-Cgas. The shNC is control. The cells were unstimulated (US) or stimulated with HSV-1 (MOI = 1) as indicated for 12 hrs. (B) qRT-PCR analysis of IFNβ in IFI16-/- A549 cells transfected with si-CGAS or si-STING. The IFI16+/+ A549 cells is control. The cells were unstimulated (US) or stimulated with HSV-1 (MOI = 1) as indicated for 12 hrs. **P < 0.01 and ***P < 0.001. Data are representative of three independent experiments (mean ± SD in A and B). (TIF) [file ppat.1008079.s005.tif]

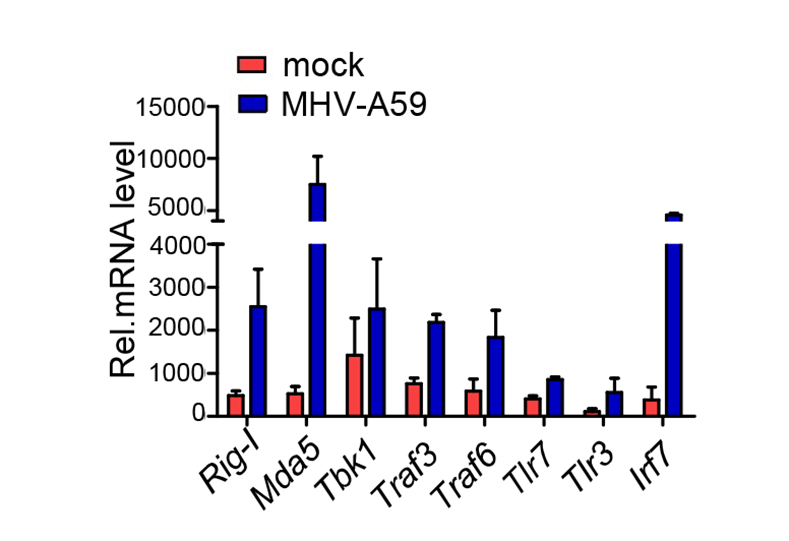

Supplement: S6 Fig — Transcriptome analysis to measure the relative expression levels of Rig-I, Mda5, Tbk1, Traf3, Traf6, Tlr3, Tlr7 and Irf7. BMDCs were infected by MHV and collected at 18 hpi. Data are representative of two independent experiments (mean ± SD). (TIF) [file ppat.1008079.s006.tif]

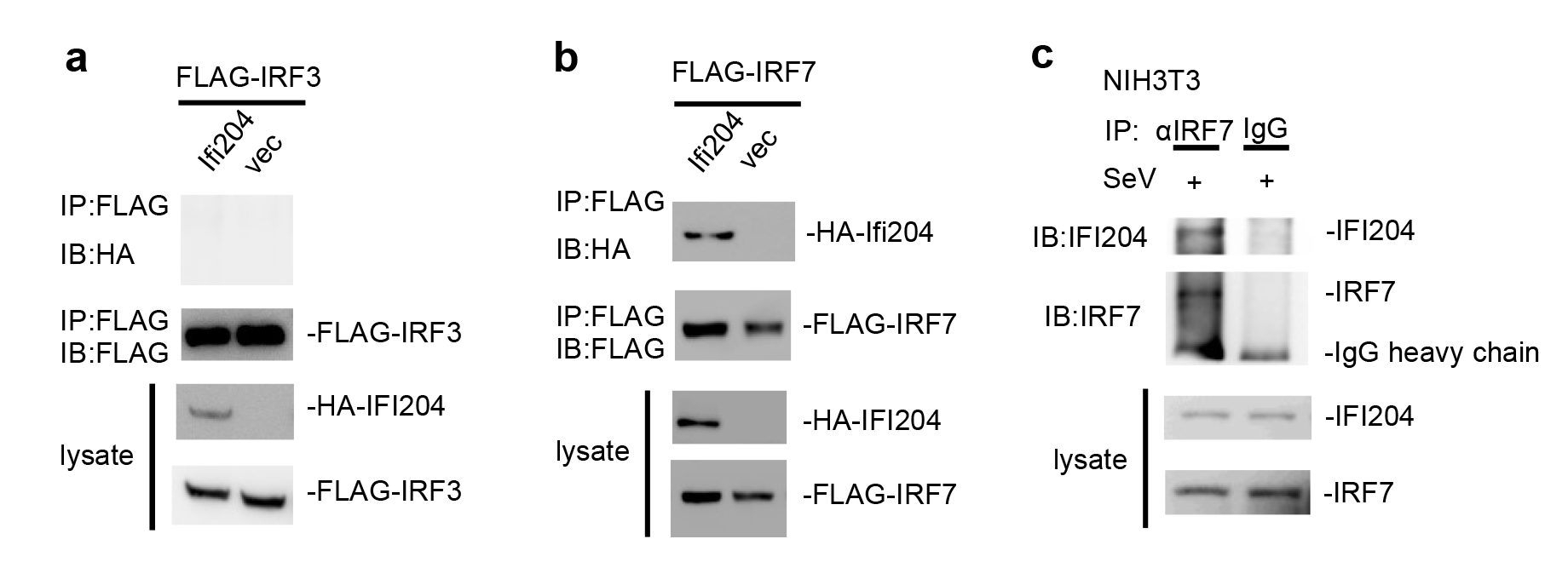

Supplement: S7 Fig — Co-immunoprecipitation and immunoblot analysis of IFI204 and IRF3 (A) or IRF7 (B and C). (A and B) HEK293T cells were co-transfected with plasmids encoding HA-IFI204 or its vector (vec) as control and Flag-IRF3 (A) or Flag-IRF7 (B) as indicated. Cell lysate was immunoprecipitated with anti-Flag and analyzed by immunoblot with anti-Flag or anti-HA antibodies. (C) NIH3T3 cells were infected with SeV for 8 hrs. Cell lysate was immunoprecipitated with anti-IRF7 antibody or control IgG, and analyzed by immunoblotting with anti-IRF7 or anti-IFI204 antibodies to detect the association of endogenous proteins. (TIF) [file ppat.1008079.s007.tif]

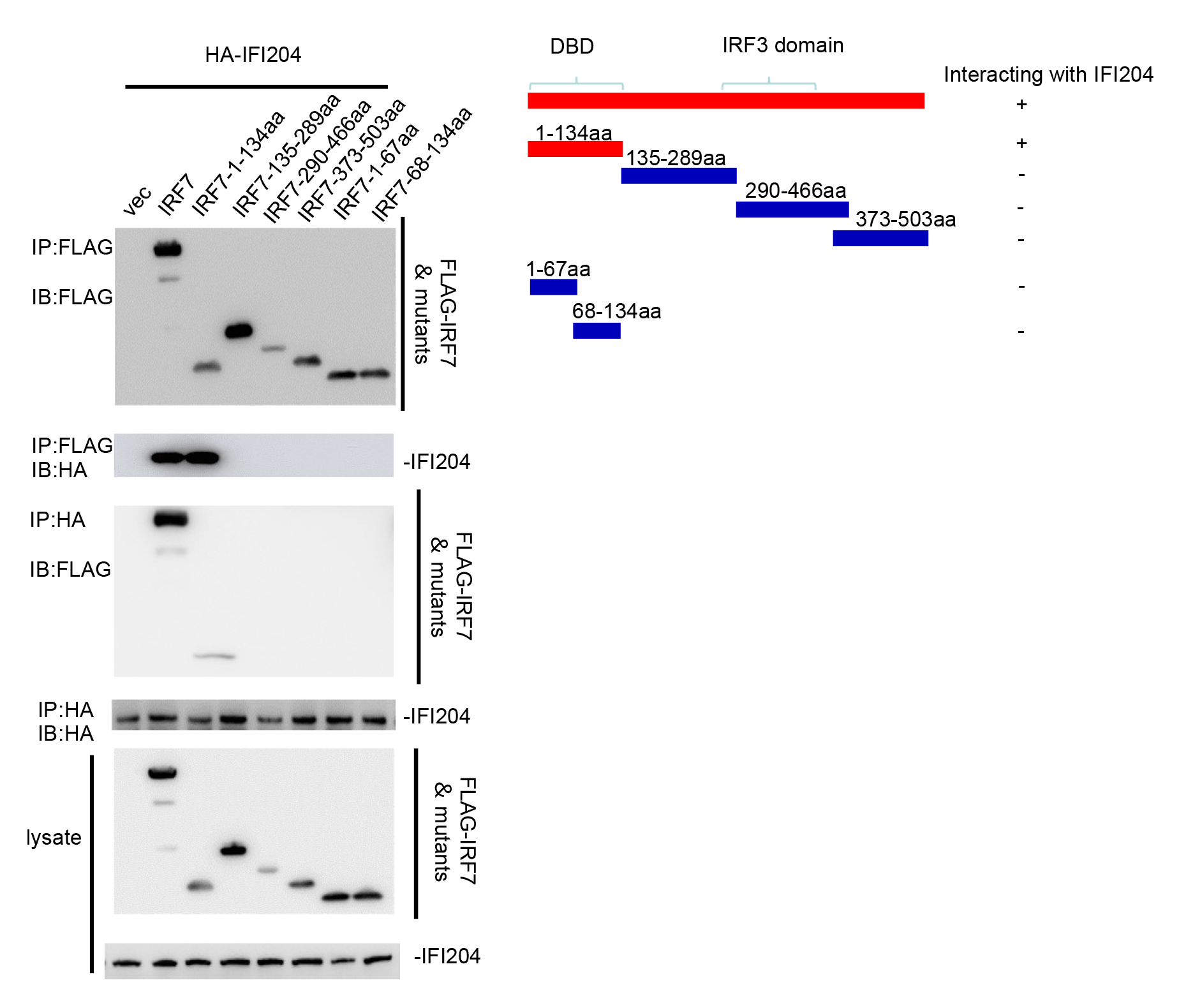

Supplement: S8 Fig — Co-immunoprecipitation and immunoblot analysis of HEK293T cells, which were transfected with plasmids encoding HA-IFI204 and Flag-IRF7 or its mutants for 24 hrs. Cell lysate was analyzed by immunoblot with anti-Flag and anti-HA antibodies. Schematic diagram to show the regions of IRF7 interacting with IFI204 (right panel). (TIF) [file ppat.1008079.s008.tif]

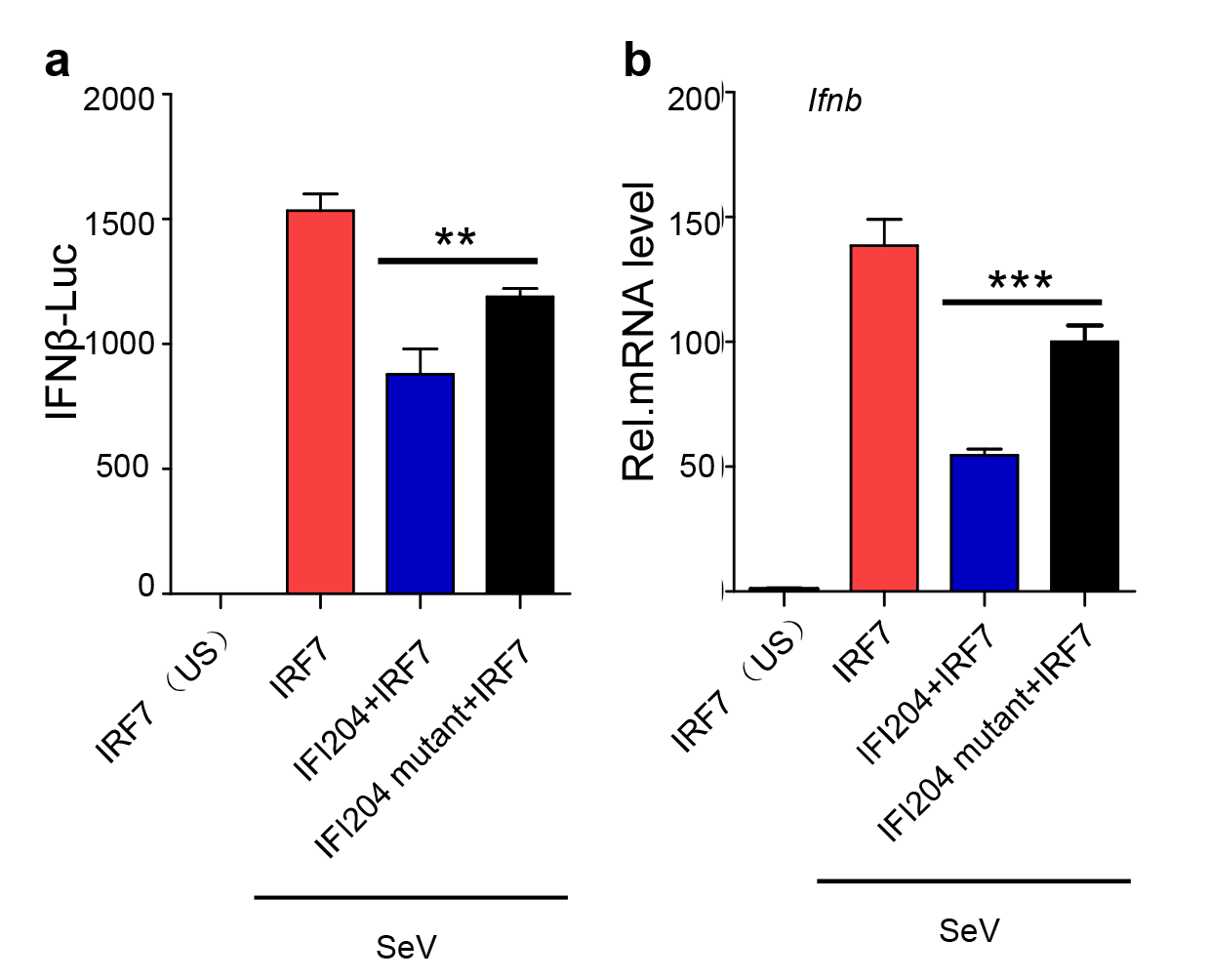

Supplement: S9 Fig — (A) Dual luciferase assays for analyzing the promoter activity of IFNβ in Irf3−/−Irf7−/− MEFs co-transfected with 200 ng IFNβ promoter luciferase reporter plasmid, 250 ng IRF7, IFI204 or IFI204 mutant as indicated. The Renilla expression plasmid (pRL-TK, 10 ng) was co-transfected as an internal control. 24 hrs post-transfection, cells were unstimulated (US) or stimulated with SeV for 12 hrs. (B) qRT-PCR analysis of Ifnb in Irf3−/−Irf7−/− MEFs co-transfected with IRF7 and IFI204 or IFI204 mutant as indicated. 24 hrs post-transfection, cells were unstimulated (US) or stimulated with SeV for 12 hrs. **P < 0.01 and ***P < 0.001. Data are representative of three independent experiments (mean ± SD in A and B). (TIF) [file ppat.1008079.s009.tif]

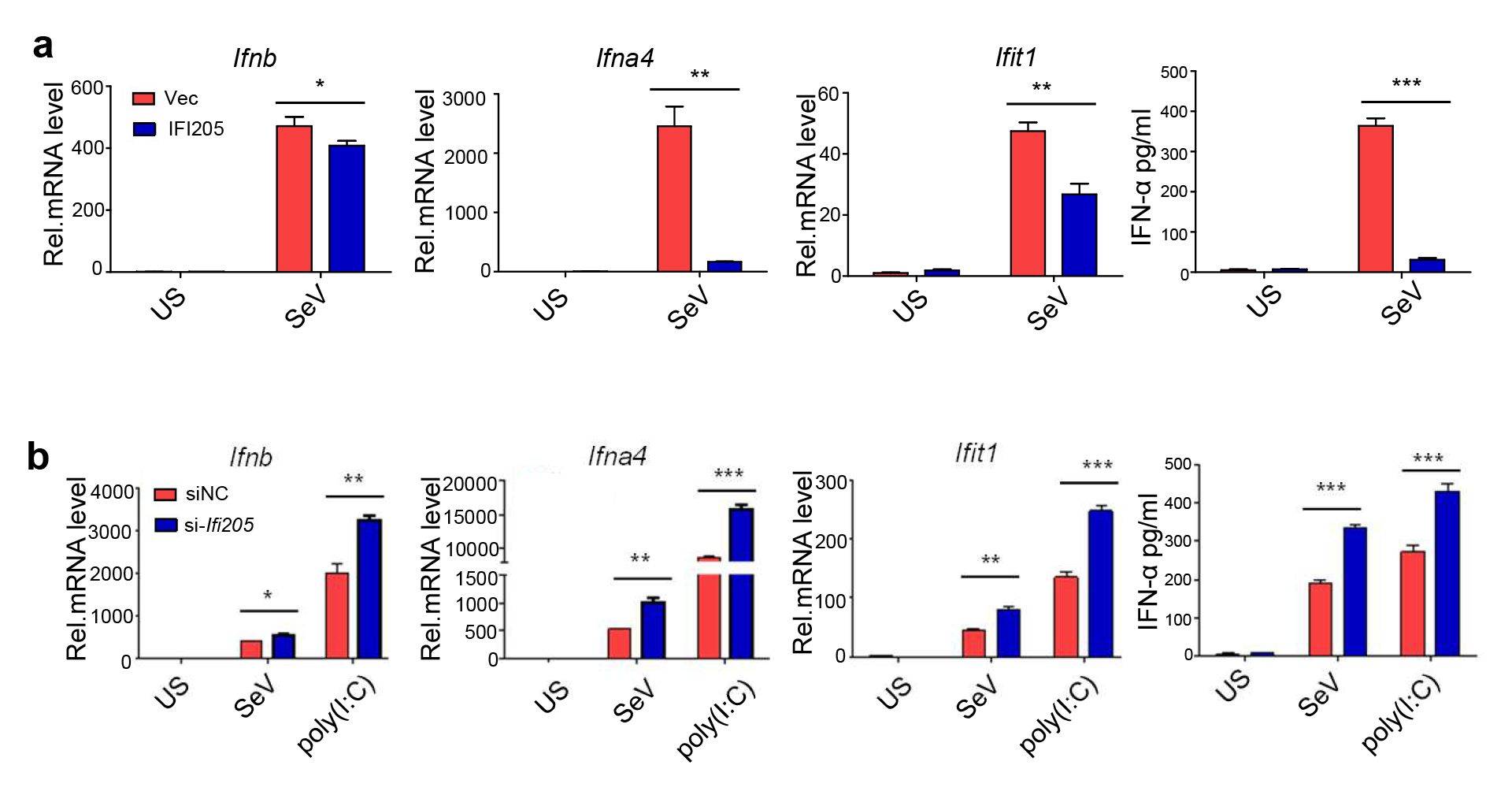

Supplement: S10 Fig — (A) qRT-PCR analysis of Ifnb, Ifna4, Ifit1 and ELISA analysis of IFNα in BMDCs stably expressing IFI205 or its vector (Vec) as negative control. BMDCs were infected by lentivirus-IFI205 or lentivirus-vector (Vec), respectively. The cells were unstimulated (US) or stimulated by SeV for 8 hrs as indicated. (B) qRT-PCR analysis of Ifnb, Ifna4, Ifit1 and ELISA analysis of IFNα in NIH3T3 cells transfected with si-Ifi205 and siNC as negative control. The cells were unstimulated (US) or stimulated by SeV or poly(I:C) for 8 hrs as indicated. *P < 0.05, **P < 0.01 and ***P < 0.001. Data are representative of three independent experiments (mean ± SD in A and B). (TIF) [file ppat.1008079.s010.tif]
