# Supplementary material for: P200 family protein IFI204 negatively regulates type I interferon responses by targeting IRF7 in nucleus
Source: PLoS Pathog. 2019 Oct 11;15(10):e1008079. doi: 10.1371/journal.ppat.1008079 (PMC6818788; doi:10.1371/journal.ppat.1008079)
Supplement: S1 Table — (DOCX) [file ppat.1008079.s011.docx]

|  | Forward 5’-3’ | Reverse 5’-3’ |
| --- | --- | --- |
| pCAGGS-Ifi204 | ACCCTCGAGGTGAATGAATACAAGAGAATT | TCTGCTAGCTCACTTTCTAGCATTGATGACC |
| pCAGGS-Ifi209 | ACCCTCGAGGTGAATGGTGAATGAATACAA | TCTGCTAGCTCACTCAATTCTTAGCTTTGAT |
| pCAGGS-Ifi205 | ACCCTCGAGATGGAGAATGAATATAAGAGA | TCTGCTAGCTCACTGGACAGTTGATGCTTC |
| pCAGGS-Mndal | ACCCTCGAGGTGAATGGCTGAATACAAGAAA | TCTGCTAGCTCACTTAGACGTGATAGTCTGG |
| pCAGGS-  Ifi16 | ACCCTCGAGGTGAATGGGAAAAAAATACAA | TCTGCTAGCTCACTTAGAAGAAAAAGTCTGG |
| pCAGGS-Mnda | ACCCTCGAGGTGAATGGTGAATGAATACAA | TCTGCTAGCTCACTCAATTAACATTCATTGG |
| pCAGGS-Aim2 | ACCCTCGAGGTGAATGGAGAGTAAATACAA | TCTGCTAGCTCACTTAGACCAGTTGGCTTGA |
| pGEX-4T-1-Ifi204 | CGTGGATCCATGGTGAATGAATACAAGAGAATT | CGAGTCGACCCGGTCACTTTCTAGCATTGATGACCTG |
| pMAL-CX2-Irf7 | TCC TCTAGAGCTGAAGTGAGGGGGGTCCA | GCCAAGCTTTCAAGGCCACTGACCCAGGTC |
| IRF7Δ247-467aa | CCCAGCCCCGAGGGTGTGTCTTCCCTGGA | CACACCCTCGGGGCTGGGGGTCGTCTCTA |

**S1 table. Primers for PCR**
