# Supplementary material for: P200 family protein IFI204 negatively regulates type I interferon responses by targeting IRF7 in nucleus
Source: PLoS Pathog. 2019 Oct 11;15(10):e1008079. doi: 10.1371/journal.ppat.1008079 (PMC6818788; doi:10.1371/journal.ppat.1008079)
Supplement: S2 Table — (DOCX) [file ppat.1008079.s012.docx]

**S2 table. Primers for mRNA Quantification**

|  | Forward 5’-3’ | Reverse 5’-3’ |
| --- | --- | --- |
| M-GAPDH | CGACTTCAACAGCAACTCCCACTCTTCC | TGGGTGGTCCAGGGTTTCTTACTCCTT |
| M-IFN-β | CCGAGCAGAGATCTTCAGGAA | CCTGCAACCACCACTCATTCT |
| M-IFN-α4 | AAGCCTGTGTGATGCAGGAA | TGGTTGAGGAAGAGAGGGCT |
| M-IFN-α6 | AGCTACTGGTCAACCTGCTCTCTAG | CCAGGAGTGTCAAGGCTTTCTT |
| H-GAPDH | ATGACATCAAGAAGGTGGTG | CATACCAGGAAATGAGCTTG |
| H-IFN-β | AGGACAGGATGAACTTTGAC | TGATAGACATTAGCCAGGAG |
| H-IFN-α4 | TTCAATCTCTTCAGCACAG | CTTCCAGGTCATTCAGTT |
